# Revisiting the Myths of Protein Interior: Studying Proteins with Mass-Fractal Hydrophobicity-Fractal and Polarizability-Fractal Dimensions

**DOI:** 10.1371/journal.pone.0007361

**Published:** 2009-10-16

**Authors:** Anirban Banerji, Indira Ghosh

**Affiliations:** 1 Bioinformatics Centre, University of Pune, Pune, India; 2 School of Information Technology, Jawaharlal Nehru University, New Delhi, India; Massachusetts Institute of Technology, United States of America

## Abstract

A robust marker to describe mass, hydrophobicity and polarizability distribution holds the key to deciphering structural and folding constraints within proteins. Since each of these distributions is inhomogeneous in nature, the construct should be sensitive in describing the patterns therein. We show, for the first time, that the hydrophobicity and polarizability distributions in protein interior follow fractal scaling. It is found that (barring ‘all-α’) all the major structural classes of proteins have an amount of unused hydrophobicity left in them. This amount of untapped hydrophobicity is observed to be greater in thermophilic proteins, than that in their (structurally aligned) mesophilic counterparts. ‘All-β’(thermophilic, mesophilic alike) proteins are found to have maximum amount of unused hydrophobicity, while ‘all-α’ proteins have been found to have minimum polarizability. A non-trivial dependency is observed between dielectric constant and hydrophobicity distributions within (α+β) and ‘all-α’ proteins, whereas absolutely no dependency is found between them in the ‘all-β’ class. This study proves that proteins are not as optimally packed as they are supposed to be. It is also proved that origin of α-helices are possibly not hydrophobic but electrostatic; whereas β-sheets are predominantly hydrophobic in nature. Significance of this study lies in protein engineering studies; because it quantifies the extent of packing that ensures protein functionality. It shows that myths regarding protein interior organization might obfuscate our knowledge of actual reality. However, if the later is studied with a robust marker of strong mathematical basis, unknown correlations can still be unearthed; which help us to understand the nature of hydrophobicity, causality behind protein folding, and the importance of anisotropic electrostatics in stabilizing a highly complex structure named ‘proteins’.

## Introduction

A student of protein structure is constantly reminded of several myths prevalent in this paradigm. He (she at any rate) studies that the globular proteins are so compactly packed that their interior mimics that of solids[1], but finds it a bit irreconcilable with reports of inhomogeneous packing[2] in protein interior and presence of cavities therein[3]. He learns about ‘hydrophobic core’ and its immense importance in making the primary sequence fold the way it does[4], but a mapping between exact amount of hydrophobicity necessary to make a certain amount of mass fold in any of the SCOP(Structural Classification of Proteins) classes, remains elusive to him. He learns that the dielectric properties of proteins are central to their stability and activity[5] but fails to find a consistent framework that relates polarizability with the bulk dielectric behavior on statistically significant number of cases. To address these myths and many more concerning structural properties of protein interior, we chose to study the inhomogeneous distributions [6] of mass, hydrophobicity and polarizability with non-integer Hausdorff-Besicovitch dimension (commonly called ‘fractal dimension’(FD)).

Although many a successful attempts have been made over three decades to describe various protein structural properties with fractal dimension based constructs; still, questions like “why not radius of gyration?”, “why not (good old) density?”, “why fractals?” - float around. Hence, there's necessity to clarify these doubts before delving into the depth of the present work. Here we present a series of facts to prove the apt nature of fractal dimension based measures in describing protein structure and protein stability.

Recent works have described proteins as ‘complex systems’[7], [8] and as ‘deformable polymers’[9]. The mesoscopic nature of protein structures has been reported by crystallographers too [10]. We know that native structures of proteins are known to be thermally stable; but at the same time, these native structures can undergo (large) fluctuations to ensure proper functioning of proteins [11], [12]. A compact object description of proteins (characterized by small amplitude vibrations and by a Debye density of low frequency modes) cannot account for such behavior of them [13]. Indeed the non-constancy of distance between any two atoms (**|r_i_−r_j_| ≠ Constant**) in any biologically functional protein can easily be verified with simplest of computer programs. Furthermore, it has been found recently that proteins exist in a state of ‘self organized criticality’[14], [15]. Along with all these, recent [6] and previous [2] characterizations of inhomogeneous distributions of mass and hydrophobicity merely serve to complicate an effort to construct a general and unambiguous scheme for description of protein interior.

Many geometrical and biophysical constructs are proposed over the years to model the multifaceted nature of protein structural parameters. Many of them are useful (and easily understandable too). Amongst them Radius Of Gyration(ROG) became known as an extremely helpful measure that could easily relate mass of any object with the size and shape of it. Originally defined for rigid bodies (where (**|r_i_−r_j_|  =  Constant**)) in the paradigm of classical mechanics[16], it found its use in the realm of polymers[17]. Later on, ROG found extensive use in protein mass-structure-shape related studies; so much so, that we use it (almost) as a benchmark property whenever we deal with any problem in the aforementioned realm (the present study is no exception either). Having said that, one may notice that the time-dependent, temperature-dependent and context-dependent nature of ROG in proteins is well documented too [8], [18]–[25]. Sensitivity of ROG on all these biophysical and/or biochemical properties, dents the profile of it to be considered as a consistent and robust marker to describe mass distribution within a given shape boundary of proteins. Indeed several studies, from time to time, have reflected upon the drawbacks and limitations of applying radius of gyration on proteins, from numerous perspectives [6], [21], [26], [27].

Another classical measure, density of the proteins can be calculated by radially partitioning the protein interior in a series of concentric shells and then measuring mass and (separately) hydrophobicity for every shell volume of a protein. (In fact this simple (mass/volume) scheme can be improved by normalizing it suitably as: (mass/volume/number of atoms)). However, as a recent study [6] proved; density, as a single valued measure of mass-packing and/or hydrophobicity-packing can be a bit involved to obtain than one expects, (a protein needs to be radially partitioned in order to calculate the density in each of the shells, before taking an average of these densities). Even if such troubles are taken, various coarse-graining operations during density calculation (while fixing the radial width of interior partitions, while averaging to obtain the final value) might anytime account for some loss of information. Apart from all that, (perhaps) most importantly, the measure ‘density’ does not possess the capability to view proteins as nonlinear complex systems, as have been asserted (from various perspectives) in recent studies [28]–[30].

Hence, we find ourselves in a conundrum where we want to describe a nonlinear object (that is protein), marked by ‘complex systems’ like biophysical properties (innate mesoscopic nature; inhomogeneous, nonlinear behaviors of structural parameters; ‘self organized criticality’ etc…); and the popular markers available for the job (namely, ROG and density), are perhaps not the best ones to epitomize the complexity that they attempt to describe. To come out of this quandary, we must be honest in our attempt to describe proteins as they are. In this context, remembering the famous quote : “Everything should be made as simple as it is, but not simpler” - Einstein; might help. Keeping this quote in mind, we adopted an approach to study protein interior that describes the inhomogeneous, nonlinear behaviors of protein structural parameters with self similarity prevalent amongst them. Indeed many previous studies on this topic (a dreadfully undersized representation can be found from references 13, [31]–[39]) had hinted that with an objective quantification of self-similarity, we can decipher the hidden symmetry that connects global patterns of macroscopic properties in proteins (say hydrophobicity distribution, polarizability distribution etc..) with the local (atomic) interactions that produce them.

In many a cases, but not always, self-similarity (geometric or statistical) is demonstrated by objects characterized by Hausdorff-Besicovich dimension (commonly called as ‘fractal dimension’). Fractal dimension (FD) can only be calculated for objects who are described by non-integer dimensions and who have self-similarity (a straight line is perfectly self-similar, but it does not have a FD; because it is characterized by topological dimension  =  1). Way back in 1982 [40], it was reported (from crystallographic data) that the backbone of myoglobin structure meanders through space in such a way that it's FD was found to be (1.66±0.04). This is very close to the theoretical value 5/3 associated with a self-avoiding random walk (SAW-3) in three-dimensional Euclidean space (**E_3_**) [40]. A straight backbone of myoglobin would have yielded dimension d = 1; whereas, if the backbone had touched every point of a lattice in **E_3_**, d would have been 3. This proved that FD can be considered as a reliable tool to extract a pattern or a regularity hidden within the irregularity of protein biophysical properties.

Having established the reason behind resorting to FD based framework, we turn our focus on the present study. Although very many studies have been performed with FD, present work assumes immense significance because of a multitude of reasons. First, it (the present work) detects the invariant patterns in variables of innately inhomogeneous nature; namely mass, hydrophobicity and polarizability distributions in protein interior and measures them with respective FD values (unambiguous, single-valued, objective markers). Second; being an integrative framework it has several inherent advantages. The shape of any protein can safely be overlooked in such a paradigm. On the other hand, various dependencies, viz. hydrophobicity packing on mass packing (and vice-versa), polarizability distribution and mass packing (and vice versa), hydrophobicity packing and polarizability distribution (and vice versa); can readily be inferred with numerical magnitudes; - all with a single run of a simple program. Third, since the scope of the present study involves all the major structural classes across a huge set of structurally aligned dataset of thermophilic and mesophilic proteins, numerous new findings regarding protein stability, packing, latent nature of biophysical properties within secondary structures etc.. - could be unearthed. These results will certainly help the new-age protein engineering and will add enormous clarity to our present understanding of protein interior. Fourth, fruitful extension of a recently proposed concept [6], the hydrophobic center (HC) of a protein (a new way to describe centrality in protein interior) is achieved here by quantifying the extent of space-filling due to hydrophobicity, keeping HC as the origin. This description of hydrophibicity distribution within proteins is inherently more honest because here the space-filling nature of hydrophobicity is described from the reference frame of its own (instead of being described from mass-distribution-centric perspective). HC describes protein's centrality by providing us with an idea as to where, in the interior of the protein, the entire effect of hydrophobicity due to all its atoms may be assumed to be concentrated. The present framework was necessary because we wanted to study the exact contribution of hydrophobicity and polarizability separately, in ensuring protein's stability (high magnitude of interior hydrophobicity content or low magnitude of polarizability, or the presence of both, lends stability to the structure of any protein). Since both hydrophobicity and polarizability are emergent statistical properties, a robust yet sensitive marker for them should be statistical in nature. Hence the use of FD to represent both was indispensable.

‘Mass Fractal Dimension’ is a generic term that marks the degree of space-filling ability of the property concerned, within any protein. A large magnitude of ‘Mass Fractal Dimension’ corresponds to more extent of space-filling, whereas a small magnitude of it symbolizes significant amount of empty space, with respect to the extent of effect of the property under consideration. Mass distribution of proteins was successfully modeled with Mass-FD recently [39], [41]. Mass-FD (MFD) can capture the entire spectrum of (time-dependent, temperature-dependent and context-dependent) fluctuations in internal motions of a protein. The success of aforementioned studies had prompted us to explore the existence of symmetry of scale invariance in the organization of three prominent (global) components that can describe protein interior, viz. mass, hydrophobicity and polarizability; - simultaneously. Obtained results of such examination, if noteworthy, may imply that the distribution of biophysical properties that govern protein folding and protein stability in general, can as well be scale invariant; and hence, self-similar in nature. Hence a systematic analysis was performed on a statistically significant population of thermophilic and mesophilic proteins across all four major classes of SCOP[42], without imposing any artificial mathematical construct on the biological unit coordinate information provided by the PDB(Protein Data Bank) [43].

Observing anomaly in scales of residue hydrophobicity[44], we chose to work with the ‘atomic hydrophobicity’ magnitudes[45]. Since any protein can be considered as an ensemble of atoms with positive or negative hydrophobic nature, using the residue-specific atomic hydrophobicity magnitudes, we could calculate the Hydrophobic Center (HC) in the same way as we had calculated center of mass of it. To derive a quantitative description of hydrophobic compactness of the protein, Hydrophobic-FD (HFD) was calculated in the same manner as MFD. In order to compare and contrast the two schools of quantifying hydrophobic compactness, Hydrophobic-ROG (H-ROG) (to describe the overall spread of hydrophobicity within a protein) and hydrophobic-FD (HFD) were calculated. While H-ROG had numerically quantified the hydrophobic compactness (albeit, treating proteins as compact solid), HFD characterized the same considering the symmetry of self-similarity in hydrophobicity distribution within proteins. The present work provides a scope to compare the two schools by presenting the results obtained from them, alongside each other.

Relationship between mass, volume and polarizability of it isn't a simple one (an account of intricate dependencies of various parameters, in the context of protein electrostatics can be found from [46]). FD-based schemes, owing to their ability to detect scale-invariance provide the template for an ideal integrative scheme that can connect atomic cloud dispositions to macroscopic polarizability. Thus approaching from aforementioned logic, representing all the 20 amino acids by their intrinsic polarizabilities[47], Polarizability-FD (PFD) was calculated for every protein. A low magnitude of protein PFD signifies less amount of polarizability within the protein, which in turn implies a small magnitude of dielectric constant; which suggests the presence of a conducive environment for electrostatic interactions. Although several polarization models have been suggested [48], extending them to the realm of proteins can be formidable. In contrast, the present procedure of characterizing polarizability distribution is biophysically reliable (the basis of it is provided by a scheme [47] that incorporates nuclear intrinsic polarizabilities. It is therefore, different from an earlier model [49], which relied upon calculation of electronic polarizabilities of residues, based on local dielectric constants of proteins). As a result, a consistent scheme that associates microscopic polarizabilities to macroscopic dielectric behavior is constructed.

## Results

HC and CM(Center of Mass) didn't overlap on each other, although a consistent trend could be observed in their residing very close to each other (<3.5 Å). This observation tends to suggest that for all the proteins, the point in their interior where the effect of their entire mass content can be supposed to be concentrated (CM), happens to be in close proximity with the point where the effect of their entire hydrophobicity content can be supposed to be concentrated (HC).

### Nature of MFD, HFD and PFD

MFD is found to be maximum (2.37) amongst the α/β thermophilic proteins and minimum (2.18) amongst all-β mesophilic proteins (**Table 1**), implying the presence of a maximum amount of vacant space within later. On the other hand, a maximum magnitude of MFD for α/β proteins explains the reason behind slowest folding rate in them, as reported recently [50], (this is easy to understand; if **M_1_** and **M_2_** denote the mass content of two proteins, and if **M_2_ > M_1_**, it takes more time to pack **M_2_** in any scaffold of shape, compared to the time taken to pack **M_1_**). The general trend of high magnitude of HFD (**Table 2**), can be attributed to the fact that hydrophobic residues are generally well conserved during evolution [51], [52]. A low magnitude of PFDs across 4 major SCOP classes confirmed the long-held myth of protein interior with low dielectric constant and explains why charge burial in the protein interior is so prohibitive and why a point mutation that introduces net charges or dipoles deep within the interior can destabilize the protein. Extremely low PFDs, 2.04 and 2.05, were observed in all-α mesophilic and all-α thermophilic proteins, respectively (**Table 3**).

**Table 1:** ^†^ Comparison of Mass-FDs (Mean ± Standard-Deviation). Maximum values in bold and Minimum values in bold & italics are shown in each tables.

Thermophilic	Thermophilic	Thermophilic	Thermophilic
α/β Mass-FD	α+β Mass-FD	All-β Mass-FD	All-α Mass-FD
**(2.37±0.16)**	(2.28±0.15)	(2.25±0.10)	(2.33±0.20)
Mesophilic	Mesophilic	Mesophilic	Mesophilic
α/β Mass-FD	α+β Mass-FD	All-β Mass-FD	All-α Mass-FD
(2.29±0.17)	(2.18±0.15)	***(2.18±0.13)***	(2.25±0.21)

**Table 2 pone-0007361-t002:** ^†^. Comparison of Hydrophobicity-FDs (Mean ± Standard-Deviation).

Thermophilic	Thermophilic	Thermophilic	Thermophilic
α/β Hydroph-FD	α+β Hydroph-FD	All-β Hydroph-FD	All-α Hydroph-FD
**(2.43±0.14)**	(2.34±0.14)	(2.35±0.09)	(2.30±0.21)
Mesophilic	Mesophilic	Mesophilic	Mesophilic
α/β Hydroph-FD	α+β Hydroph-FD	All-β Hydroph-FD	All-α Hydroph-FD
(2.33±0.15)	***(2.22±0.15)***	(2.29±0.12)	(2.23±0.20)

**Table 3 pone-0007361-t003:** ^†^. Comparison of Polarizability-FDs (Mean ± Standard-Deviation).

Thermophilic	Thermophilic	Thermophilic	Thermophilic
α/β Polarizability-FD	α+β Polarizability-FD	All-β Polarizability-FD	All-α Polarizability-FD
(2.10±0.04)	(2.10±0.05)	(2.08±0.03)	(2.05±0.02)
Mesophilic	Mesophilic	Mesophilic	Mesophilic
α/β Polarizability-FD	α+β Polarizability-FD	All-β Polarizability-FD	All-α Polarizability-FD
**(2.14±0.06)**	(2.11±0.05)	(2.12±0.05)	***(2.04±0.02)***

**Figure 1:**
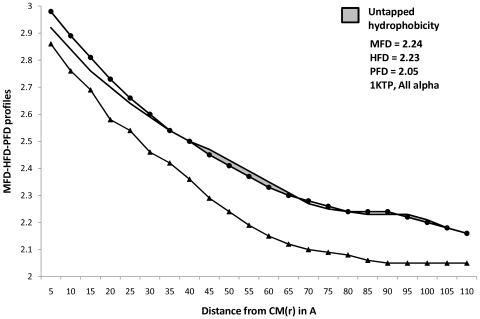
Profiles of MFD, HFD, PFD and Untapped Hydrophobicity in all-α proteins. Magnitudes of Mass, Hydrophobicity and Polarizability are plotted simultaneously for the (randomly chosen) all-α protein ‘1ktp’. Linear portions of in their profiles denote the MFD, HFD and PFD magnitudes. [+(HFD-MFD] assumes a negative magnitude for all-α proteins. In the present case, ‘Untapped Hydrophobicity’ ([+(HFD-MFD]) is given by : [+(HFD-MFD]  =  [(2.23–2.24)] space-filling unit  =  [- 0.01] space-filling unit. Negative magnitude of ‘untapped hydrophobicity’ could only be observed in all-α proteins. — • — : MFD line, only the linear portion of it (parallel to X-axis) provides the MFD value. ——— : HFD line, only the linear portion of it (parallel to X-axis) provides the HFD value. —▴— : PFD line, only the linear portion of it (parallel to X-axis) provides the PFD value.

Profiles of MFD, HFD and PFD for representative proteins from four major structural classes are plotted in figure-1 to figure-4. Stretches of these profiles can be observed to be parallel to abscissa. These (parallel) stretches represent scaling ranges where ordinate variation is invariant with respect to variation in abscissa; in other words, they correspond to scaling ranges where ‘scale-invariance’ in the profiles of MFD, HFD and PFD exist. Magnitude of the ordinate corresponding to this ‘scale-invariant’ stretch of profile is defined as the fractal dimension of the profile under consideration. While **Table- 1** **–** [Table pone-0007361-t002] [Table pone-0007361-t003] **4** (discussed later) compared the differences in the magnitude of HFD and MFD, the general trend of their profiles could not be understood from merely these magnitudes. Furthermore, tabular magnitudes could not reveal the scaling ranges on which FD magnitudes were observed. Both these requirements could be addressed simultaneously by plotting the trends in MFD, HFD and PFD profiles. **Fig- 1** **–** [Fig pone-0007361-g002] [Fig pone-0007361-g003] **4**, therefore enabled us to compare between the trends that describe MFD and HFD over different scaling ranges. Hence a more comprehensive comparison between HFD and MFD magnitudes could be performed by observing the entrapped area between them, for any protein belonging to any structural class.

**Figure 2 pone-0007361-g002:**
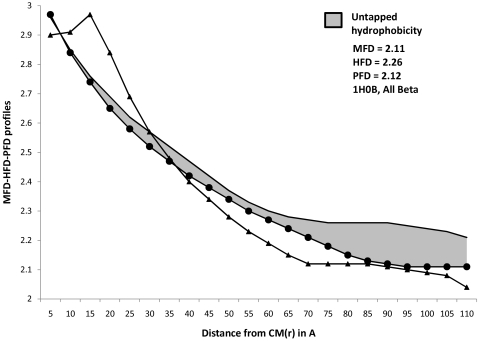
Profiles of MFD, HFD, PFD and Untapped Hydrophobicity in all-β proteins. Magnitudes of Mass, Hydrophobicity and Polarizability are plotted simultaneously for the (randomly chosen) all-β protein ‘1h0b’. Linear portions of in their profiles denote the MFD, HFD and PFD magnitudes. [+(HFD-MFD] assumes the maximum magnitude for all-β proteins. In the present case, ‘Untapped Hydrophobicity’ ([+(HFD-MFD]) is given by : [+(HFD - MFD]  =  [(2.26−2.11)] space-filling unit  =  [+ 0.15] space-filling unit. The maximum magnitude of ‘untapped hydrophobicity’ could be observed in all-β proteins. — • — : MFD line, only the linear portion of it (parallel to X-axis) provides the MFD value. ——— : HFD line, only the linear portion of it (parallel to X-axis) provides the HFD value. —▴— : PFD line, only the linear portion of it (parallel to X-axis) provides the PFD value.

**Figure 3 pone-0007361-g003:**
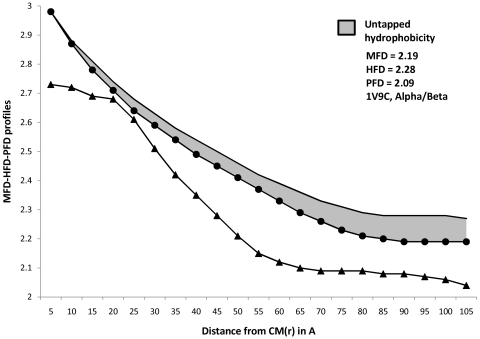
Profiles of MFD, HFD, PFD and Untapped Hydrophobicity in α/ β proteins. Magnitudes of Mass, Hydrophobicity and Polarizability are plotted simultaneously for the (randomly chosen) α/β protein ‘1v9c’. Linear portions of in their profiles denote the MFD, HFD and PFD magnitudes. In the present case, ‘Untapped Hydrophobicity’ ([+(HFD-MFD]) is given by : [+(HFD - MFD]  =  [(2.28−2.19)] space-filling unit  =  [+ 0.09] space-filling unit. [+(HFD-MFD] magnitude for α/β proteins lie in between that of (all-β) and (all-α). — • — : MFD line, only the linear portion of it (parallel to X-axis) provides the MFD value. ——— : HFD line, only the linear portion of it (parallel to X-axis) provides the HFD value. —▴— : PFD line, only the linear portion of it (parallel to X-axis) provides the PFD value.

**Figure 4:**
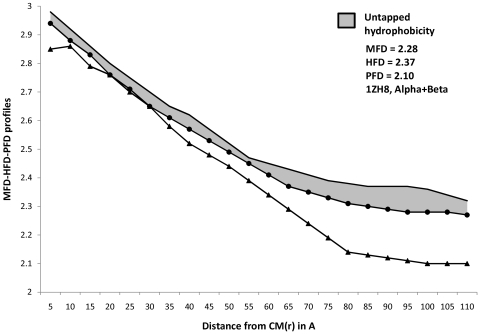
Profiles of MFD, HFD, PFD and Untapped Hydrophobicity in α+β proteins. Magnitudes of Mass, Hydrophobicity and Polarizability are plotted simultaneously for the (randomly chosen) α+β protein ‘1zh8’. Linear portions of in their profiles denote the MFD, HFD and PFD magnitudes. In the present case, ‘Untapped Hydrophobicity’ ([+(HFD-MFD]) is given by : [+(HFD - MFD]  =  [(2.37−2.28)] space-filling unit  =  [+ 0.09] space-filling unit. [+(HFD-MFD] magnitude for α+β proteins lie in between that of (all-β) and (all-α). — • — : MFD line, only the linear portion of it (parallel to X-axis) provides the MFD value. ——— : HFD line, only the linear portion of it (parallel to X-axis) provides the HFD value. —▴— : PFD line, only the linear portion of it (parallel to X-axis) provides the PFD value.

**Table 4:** † **.** Mass-Hydrophobicity-Polarizability Fractal Dimension values Across four major SCOP classes.

α/β Mass-FD	α+β Mass-FD	All-β Mass-FD	All-α Mass-FD
Composite set	Composite set	Composite set	Composite set
**(2.33±0.17)**	(2.24±0.16)	***(2.21±0.12)***	(2.29±0.21)
α/β Hydroph-FD	α+β Hydroph-FD	All-β Hydroph-FD	All-α Hydroph-FD
Composite set	Composite set	Composite set	Composite set
**(2.38±0.15)**	(2.29±0.16)	(2.32±0.11)	***(2.26±0.21)***
α/β Polarizability-FD	α+β Polarizability-FD	All-β Polarizability-FD	All-α Polarizability-FD
Composite set	Composite set	Composite set	Composite set
**(2.12±0.05)**	(2.10±0.05)	(2.10±0.04)	***(2.05±0.02)***

† NOTE : Detailed break-ups of Table-1–4 results are available on Materials S1 & S2

Careful examination of Figure-1–4, however, suggests an absence of general pattern in the scaling ranges for the magnitude of MFD, HFD and PFD across four major SCOP classes. Although, within particular classes, feeble trends in scaling ranges could be observed. To amplify this point with categorical examples, let us consider **Fig.-1**. The parallel stretches of MFD, HFD and PFD profiles for the all-α protein ‘1ktp’ (c-phycocyanin of synechococcus vulcanus at 1.6 Å) show that the scale invariance of mass and hydrophobicity within it can be observed at ∼(80–90)Å from the CM and HC respectively (since HC and CM are in proximity, we can ignore the small error introduced in the distance calculation, without any loss of generality), whereas the scale-invariance in polarizability distribution can be detected ∼(95–110)Å distance from the CM. Hence MFD and HFD could be detected at about (10–15)Å nearer to the CM of the protein, than the distance range where PFD could be detected. This trend is observed in most of the all-α proteins. Interestingly, for all-??? proteins, the situation changes. For example, let us consider **Fig.-2**. For the all-β protein ‘1h0b’ (Rhodothermus Marinus Cel12A at 1.8 Å), the scale invariance with respect to hydrophobicity can be detected at ∼(77–90) Å from the HC, whereas the same for mass can be detected at a far away (100–110)Å. Interestingly, the PFD of ‘1h0b’, can be detected at a much nearer distance, (70–85)Å from the CM. However, unlike the case for all-α proteins, scaling ranges do not always follow this pattern amongst the all-β family. Such absence of general pattern in scaling ranges within proteins is easily understandable. Being non-idealistic systems, a particular rule-based regime of scaling laws for biophysical properties of all the proteins, would have been highly improbable to conceive, at the first place.

### Correlation between number of atoms, MFD, HFD, ROG and H-ROG

Correlation studies in **Table-5** **–** [Table pone-0007361-t006] **7** describe the dependence of relevant properties on each other. From a generalized perspective, the high correlation between mass and hydrophobicity (**Table-5**) (across 4 SCOP classes, thermophilic and mesophilic alike) coupled with (rather) insignificant correlation coefficient between mass and polarizability distributions, reinforced the myth regarding hydrophobicity as the primary driving force behind protein folding. A set of strong correlations, viz. (0.91) between the MFD and ROG, (0.84) between number of atoms and MFD, (0.87) between HFD and H-ROG, and (0.78) between total number of atoms and HFD, drawn from the entire dataset (**Table-6**); vindicated the reliability and consistency of this approach. Looking at it differently, (0.09), (0.16) and (0.22) units of (hitherto unknown) information from three aforementioned correlations (respectively), implied that newer information can still be unearthed; if proteins are considered as complex mesoscopic systems, instead of being viewed as rigid bodies. The scaling of mass and hydrophobicity distribution with α+β proteins (**Table-7**) could easily be identified as one with least space-filling nature.

**Table 5:** Correlation between biophysical properties (measured with respective FDs) across four major structural classes of proteins.

Correlations between biophysical properties across SCOP classes	Correlation between MFD-HFD	Correlation between MFD-PFD	Correlation between PFD-HFD
α/β	0.98	0.12	0.10
α+β	0.97	0.45	0.39
All-β	0.96	(−0.07)	(−0.06)
All-α	0.99	0.23	0.20

**Table 6 pone-0007361-t006:** ^‡^. Correlations amongst No. of atoms, Radius Of Gyration(ROG), MFD, Hydrophobic-ROG.

Correlations across entire protein set	No. of atoms and MFD	No. of atoms and ROG	MFD and ROG	No. of atoms and HFD	No. of atoms and Hydrophobic-ROG	HFD and Hydrophobic-ROG
412 proteins	0.84	0.88	0.91	0.78	0.87	0.87

Abbreviations :

MFD : Mass Fractal Dimension; HFD : Hydrophobic Fractal Dimension.

ROG : Radius Of Gyration ; Hydrophobic-ROG : Hydrophobic-Radius Of Gyration.

**Table 7:** ‡ **.** Correlations amongst No. of atoms, Radius Of Gyration(ROG), MFD, Hydrophobic-ROG and HFD across major SCOP classes, considering the entire set of proteins.

Correlations across SCOP classes	No. of atoms and MFD	No. of atoms and ROG	MFD and ROG	No. of atoms and HFD	No. of atoms and Hydrophobic-ROG	HFD and Hydrophobic-ROG
α/β	0.87	0.90	0.91	0.82	0.90	0.86
α+β	0.79	0.82	0.91	0.73	0.82	0.87
All-β	0.91	0.92	0.91	0.81	0.91	0.84
All-α	0.85	0.86	0.90	0.80	0.85	0.88

Comment : Table-7 is a break-up of Table-6, showing contributions from each SCOP class.

‡ NOTE : A further break-up, depicting the thermophilic and mesophilic contributions to each of these classes towards all the analyzed correlations, is provided in the Materials S7.

### Existence and implication of untapped-hydrophobicity in proteins

**Figure 5:**
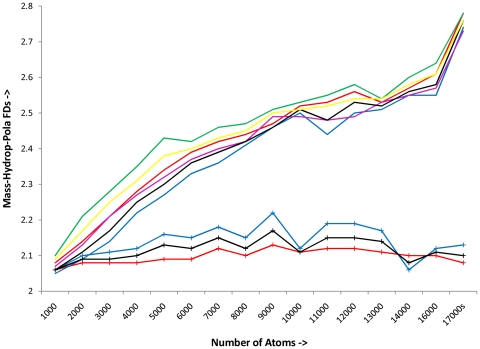
Distribution of MFD, HFD and PFD for thermophilic and mesophilic proteins. Mean magnitudes of MFD, HFD and PFD for each group of proteins (segregated by their number of atoms) are plotted. ([+(HFD-MFD]) provides an unambiguous measure of ‘untapped-hydrophobicity’, it assumes greater magnitude for thermophilic proteins(across SCOP classes) than the mesophilic proteins. Legends: Red solid line (—) **:** Distribution of Thermophilic Mass-FD Green solid line (—) **:** Distribution of Thermophilic Hydrophobic-FD Red (**+—+**) pattern **:** Distribution of Thermophilic Polarizability-FD Blue solid line (—) **:** Distribution of Mesophilic Mass-FD Pink solid line (—) **:** Distribution of Mesophilic Hydrophobic-FD Blue (**+—+**) pattern **:** Distribution of Mesophilic Polarizability-FD Black solid line (—) **:** Distribution of Mass-FD of the entire protein set. Yellow solid line (—) **:** Distribution of Hydrophobic-FD of the entire protein set. Black (**+—+**) pattern **:** Distribution of Polarizability-FD of the entire protein set. **Please consult the Supplementary Mat S3 for complete information regarding this diagram.**

**Tables- 1** **–** [Table pone-0007361-t002] [Table pone-0007361-t003] **4** reveal truly startling results where they show a consistent trend (barring all-α proteins) of higher magnitude of HFD entries (T**able-2**) with respect to the corresponding MFD entries (T**able-1**). For all-β, α/β and α+β proteins, the difference between analogous entries between T**able 2** and **Table-1** can be observed to be greater in the case of thermophilic proteins than their mesophilic counterparts; further, the absolute magnitude of MFD is invariably found to be greater in thermophilic proteins. Another distinct trend was found in lower PFD of thermophilic proteins for α/β, α+β and all-β classes, in comparison to their structurally aligned counterparts from mesophilic ensemble. However, the most striking observation from **Tables- 1** **–** [Table pone-0007361-t002] [Table pone-0007361-t003] **4**Ds. Since in all the cases (barring all-α) the HFD is greater than the MFD, it hints that certain amount of hydrophobicity is not utilized for folding (and subsequently, for packing) purposes. The extent of this ‘untapped-hydrophobicity’ can be obtained in precise terms from the detailed comparison between MFD and HFD for four SCOP classes, separately for thermophilic and mesophilic proteins, as presented in **Materials S1** **&** **S2**. By plotting MFD, HFD and PFD as functions of number of atoms, **Fig.-5** proves that the extent of unexploited-hydrophobicity is more in thermophilic proteins than in mesophilic proteins, across all the SCOP classes; because the entrapped area between HFD and MFD curve is more in the former. Observations from **Table-5** and **Table-7** shows that the composite correlation between MFD and HFD is ∼97%, while that between number of atoms and HFD is ∼79% only; whereas correlation between number of atoms and MFD is ∼86%. These correlations suggest that mass distribution within a protein scales up in similar manner as hydrophobicity does, but protein atoms do not consume the property hydrophobicity as much as they consume the mass of them; supporting the possible inference of untapped-hydrophobicity further. **Fig- 1** **–** [Fig pone-0007361-g002] [Fig pone-0007361-g003] **4**depict the differences between trends describing HFD and MFD profiles in all four protein structural classes, by the entrapped (marked) area between the profiles of them. The immense significance of the ([+(HFD-MFD)]) magnitudes, the trends that describe them, and especially, the outstanding difference between the marked area for all-β and all-α proteins are discussed later.

## Discussion

High correlation between HFD and MFD was expected. The existing knowledge base, built from four decades of protein-folding and protein-structure studies had indicated that the strength of hydrophobic effect (energy of stabilization provided by the transfer of hydrocarbon surfaces from solvent to the interior of a protein) accounts largely for the stability of folded structure of globular proteins. Our study, from a different perspective altogether, vindicated it. However, without stopping there, the present study went ahead to report some unexpected (and hitherto unreported) phenomena too.

### Interpretation(s) of ‘untapped hydrophobicity’, relating ‘untapped hydrophobicity’ to contemporary knowledge of protein structure

The revelation of untapped hydrophobicity ([+(HFD-MFD)]) suggests that the thermophilic proteins, although better packed than their structurally aligned mesophilic counterparts, are not maximally packed, as they could have been. Causality of this unexploited hydrophobicity in thermophilic proteins can be attributed to their effort in ensuring the desired functionality, while adapting to the hostile environment; a stringent requirement, which the mesophilic proteins are not subjected to. Since proteins function primarily by topographic surface recognitions, the necessary scaffold for surface topography of any protein needs to be maintained at any cost and probably that's why thermophilic proteins do not utilize the bulk of hydrophobicity that they could have used to fold in an even more compact manner. Mesophilic proteins do not possess the same quantity of hydrophobicity as their thermophilic counterparts, but they utilize the available hydrophobicity better towards their packing purpose.

Other than the aforementioned rationalization of [+(HFD-MFD)] from perspective of protein function, an in-depth explanation from structural perspective can be presented to understand the systematic trend of higher magnitude of untapped hydrophobicity in thermophilic proteins. Since kinetic energy amongst the atoms of thermophilic proteins can be expected to be greater (by quite some fold) than the same in their structurally aligned mesophilic counterparts, to ensure structural integrity in the thermophilic proteins, a higher magnitude of cumulative hydrophobicity is required. However, since the amount of mass packing is almost the same in structurally aligned proteins, to make the structure of a thermophilic protein secured, hydrophobicity needs be more space-filling as a property. More space-filling nature of hydrophobicity will ensure that every portion of the thermophilic protein space can utilize the effect of it. Therefore, [+(HFD-MFD)] assumes a higher value for the thermophilic proteins. On the other hand, for the mesophilic proteins, the necessity of the effect of hydrophobicity to reach out to all portions of the protein in order to ensure its stability is not so acute; henceforth, [+(HFD-MFD)] values for them assume (comparatively) less magnitude than the same in their thermophilic counterparts. This point of view is supported by the evidence [53] where hydrophobicity content of proteins was observed to increase during increment of temperature; implying that proteins do resort to hydrophobicity to maintain their structural integrity (when subjected to perturbations). Therefore, this marker [+(HFD-MFD)], namely the ‘untapped hydrophobicity’, can be considered a general structural feature, which quantifies the stability cushion that a protein can avail to sustain structural perturbation.

We chose to assemble proteins (irrespective of their SCOP classes) with respect to their number of atoms to plot the mean magnitudes of MFD, HFD and PFD for each of these groups as dependent variables of their number of atoms (**Fig-5** and **Materials S3**). Such depiction clearly showed that although the area entrapped between the distributions of HFD and MFD for the thermophilic proteins is more than the same for mesophilic proteins; there is, nevertheless, an amount of space entrapped between HFD and MFD distributions for mesophilic proteins too. Hence for any set of proteins, if the group MFD and HFD distributions are expressed as functions of number of atoms (x), say ö(x) and f(x) respectively; then [ I =  _x1_∫^x2^ (f(x) - ö(x))] can be expected to provide an objective measure of ‘untapped-hydrophobicity’ present in that set of proteins. For the case of any particular protein, the existence of positive (HFD – MFD) provides an unambiguous measure of such ‘untapped-hydrophobicity’. The existence of ‘untapped-hydrophobicity’ suggests clearly that the packing within the protein, although compact, is not maximal.

We are completely aware that many other interpretations of [+(HFD-MFD)] can be put forward; which, (at least apparently) might seem different than the present one, namely the ‘untapped-hydrophobicity’. For example, numerous studies have (explicitly or implicitly) pointed at the existence of a small subset of residues with critical hydrophobic profile to dominate protein folding [54]–[56]. It has also been noticed that many proteins contain cavities in their interior and probabilities of these cavities being filled up by amino acids with pronounced hydrophobic nature is non-trivial too (the so called ‘hydrophobic core’) [56]–[59]. Hence [+(HFD-MFD)] can be explained from this standpoint as well; albeit only for the specific cases where categorical information about internal cavities being filled up with hydrophobic residues are known. Hence, it is difficult to establish a general pattern of [+(HFD-MFD)] across three structural classes, merely based on this observation.

Another interpretation that relies upon existing set of knowledge about protein stability and accounts for the existence of ‘untapped hydrophobicity’ is provided in **Materials S4**.

The dramatic result in the stability profile of a protein, due to systematic replacement of amino acids (with comparable ones) in the hydrophobic core composition is a well-known fact [59], [60]. However, such studies were carried out on individual proteins under particular biophysical and biochemical boundary conditions. Our work lends a theoretical support to such experimentally obtained results and proves, in a generalized way, that it is not the mass of the atoms themselves, but the cumulative effect of hydrophobicity (space-filling nature of hydrophobicity) that governs protein stability. The quantity [+(HFD-MFD)], for a single protein, quantifies how much of space-filling does protein's hydrophobicity need to achieve with a given value of space-filling of protein's mass. Need for such accurate quantification of exact amount of mass requiring an exact magnitude of hydrophobicity to ensure protein's stability was acutely felt in some previous studies [59], [60]. Hence, the present work might be extremely useful in case of many problems (specific or general) of protein design and protein engineering.

### MFD, HFD, PFD and secondary structures

A set of minute observations leads us to have a critical look at another set of myths regarding the structural nature of origin of α-helices and β-strands. A combination of observations from **Table- 1** **–** [Table pone-0007361-t002] [Table pone-0007361-t003] **4**, shows the existence of [-(HFD-MFD)] amongst all-α class (thermophilic and mesophilic) of proteins, which contradicts the general tendency of existence of [+(HFD-MFD)]. Furthermore, the magnitude of [-(HFD-MFD)] can be observed to be more in the realm of thermophilic proteins, than mesophilic ones. A closer look at the PFD magnitudes of all-α proteins helps us resolve this anomaly. Since PFD**_all-α_** possess the lowest magnitudes in comparison to those in other SCOP classes, the most favorable environment for electrostatic interactions can be expected to be there in all-α proteins. Moreover, since the PFD for the thermophilic all-α proteins can be observed to be of the lowest amongst all the 8 classes under consideration, it explains the causality behind the second best well-packed nature of all-α proteins (next to α/β) amongst the thermophilic proteins; despite its having the least HFD.

On the other hand, the all-β proteins are found to be endowed with second highest HFD magnitudes in thermophilic and mesophilic proteins. The amount of ‘untapped-hydrophobicity’ is found to be the maximum amongst the all-β proteins; furthermore, going against the general trend, it is the all-β proteins from mesophilic organisms (and not from the thermophilics) that can be observed to contain the highest magnitude of [+(HFD-MFD)], implying clearly that the packing within all-β mesophilic proteins is farthest from being maximal. Careful observation of **Fig. 1** and **Table-1** of **Materials S1** **and** **S2** reveals the startling pattern that in 100% of moderately large all-β proteins (5000 ≤ No. of atoms <8000), the pattern of (MFD < HFD) is observed; whereas, when compared to the second decimal place, in ∼86% of small (No. of atoms <5000) all-α proteins, the conspicuous pattern of (MFD > HFD) could be observed. Remarkableness of these patterns lies in their representations by overwhelming proportions of population belonging to all-α and all-β proteins. Distribution patterns of hydrophobicity, polarizability, and ‘untapped-hydrophobicity’ within α/β and α+β proteins show no noteworthy features and can be roughly approximated by a mean distribution profile of these parameters in all-α and all-β proteins.

These findings are not entirely unexpected since a hydrophobic origin for β-sheets have been proposed by many researchers now and then [61], [62] and similarly an extremely low polarizability is associated with the origin of α-helices for a long time too [63]. However, such assertions were few and far between in nature and more importantly, did not have a full-fledged general theory to explain the causality behind the observations. The myth of hydrophobic origin of α-helices had probably originated due to a perceived concurrent occurrence of formation of α-helix and the so-called ‘hydrophobic collapse’, although the pitfalls of such formulation were pointed out recently [64].

To assess the justification and utility of the present model, it was crucial for us to compare it with an extremely popular model that describes helix-electrostatics and helix folding; namely, the Helix/Coil Transition Theory (HCTT). A detailed account of this comparison is provided in **Materials S5** ; it shows how and why the present methodology scores above the HCTT.

On a different note, the lack of conducive environment for strong electrostatic interactions helps us to understand reason behind easy profile of denaturation of β-sheet structures and all-β proteins, as reported in several experimental studies [65], [66]. The same reason explains the reason behind (conspicuous) absence of all-β classes from molten globule state of a protein [67], [68]. (Generally, proteins in vitro can be transformed into the molten globule state at low pH or in moderate concentrations of the chemical denaturants or at high temperatures[69]; all of which imply that any protein with loose electrostatic attraction in its interior (like the all-β proteins) will not be able to survive aforementioned boundary conditions).

### Comparison between the present framework and ‘binary partitioning’ scheme

Our algorithm to describe protein interior through a composite view of MFD, HFD, PFD (all measured simultaneously from a common mathematical foundation) becomes pretty handy in some other instances too. The ‘binary partitioning’ scheme [70] (compactly packed interior with high content of hydrophobicity and loosely packed exterior with low content of hydrophobicity) for proteins can describe many a cases; but it cannot describe all. While, some previous studies have documented the shortcomings of such simplified schemes [71], [72]; here we will only concentrate on the drawbacks of it from categorical perspectives. For example, in the cases involving loose interior packing [73]–[76] (pretty prevalent in the realm of helical membrane proteins), the aforementioned (binary partitioning) scheme fails. Plight of binary partitioning based protein structure studies becomes even more acute when the packing defect of protein interior carries out a random walk within a finite domain of the channel proteins [77], [78]. The inherent advantage of resorting to simultaneous calculation of MFD-HFD-PFD is that it will work as efficiently on proteins within the lipid bilayer as on the water-soluble ones. Furthermore, it will treat atoms in the highly compact zone with equal importance as the ones where large packing deficiencies occur (say interior voids or cavities). This innate power of generalization stems from the fact that fractal description of protein interior does not resort to (simplistic) rigid body description of proteins with distinguishable atoms, but describes them as “complex systems”[7], [8] with mesoscopic properties[10]. Therefore, phenomena like 'packing defect', 'deformability' etc., can be automatically taken into account, because FD based schemes do not treat them as “defects” or some kind of deviation from simplistic idealizations, but treat them as they are.

### ‘Untapped hydrophobicity’ and ‘marginal stability’ of the proteins

The present findings provide a unique perspective to look at “marginal stability” of proteins (ΔG folding ∼ 10 kcal/mol) that are reported in numerous studies from present and past [79]–[81]. It is known that evolutionary constraints of protein folding and protein structure are threefold; viz., folding must take place quickly, the folded structure must have a (reasonably) stable scaffold, and the folded structure must be able to perform a specific function [79]. While the requirement of marginal stability to ensure protein functionality was an established finding, mechanism through which hydrophobicity content of a protein can be related to its marginal stability was not exactly clear. In the light of the proposed concept of ‘untapped hydrophobicity’, all the constraints and relations described above can be explained from a coherent standpoint. If the protein under consideration had exploited the amount of available hydrophobicity in its entirety, it would have attained a perfect stability. But such perfectly stable conformation would have failed to ensure its foldability of the protein. The loss of foldability would have resulted in its not attaining the “marginal stability” [79] and would have jeopardized its function (because of its inability to transform its shape). Hence, ‘untapped hydrophobicity’ provides us with the missing link to understand how and why proteins maintain just the optimal stability in them.

### Compatibility of the FD-based (Top-Down) results with amino-acid based (Bottom-Up) results

An extremely important and enormously interesting facet of our work unfolds when one compares the magnitude of biophysical properties predicted from our algorithm, with the existing (huge) knowledge-base, regarding properties of amino acids. This assumes significance because the present work was based solely upon the statistical description of distribution of key biophysical properties in protein interior and it had never resorted to bottom-up analysis of amino acid features. But remarkably, results obtained from the present algorithm could describe the general (emergent) features of secondary structures, from a completely different perspective. For example, consider the best makers of anti-parallel β-sheets. They are Val, Tyr, Ile, Phe, Cys and Trp. Those for parallel β-sheets are Val, Ile, Phe, Leu, Tyr[82]. All of which are known for their distinct hydrophobic nature. This (along with results from a related studies [83],[84]) lends the bottom-up reasoning behind the emergent general trend of presence of maximum untapped hydrophobicity in β-sheet structures. Furthermore, similar attempts bring to fore the pattern that, polar residues tend to code for helices, while hydrophobic residues typically form β-sheets. While these known patterns were all studied from bottom-up approaches all these days, the present algorithm establishes the connection between individualistic studies on residues with holistic view of biophysics of protein interior. Establishing such connections have utilitarian benefits too. The present algorithm could extract features that would have been difficult to gather from application of (traditional) bottom-up approach (where atoms and residues are identifiable). For example, consider constructing an electrostatic profile of α-helix which has Asp and Glu. These are similarly charged residues and are expected to have similar electrostatic effects when placed in a helix; but they show very different helix propensities (Asp(Chou-Fasman propensity 101 for α-helix) and Glu (Chou-Fasman propensity 151 for α-helix))[82]. Since, these two residues have quite different conformational features, it would have been difficult to construct a holistic picture of electrostatic nature of the helix by attempting to integrate the effects due to these two. However, with our algorithm that holistic picture can be obtained without referring to the atomistic and/or residue-centric information, because it does not distinguish between atoms belonging to Asp from the atoms belonging to Glu.

### On the correlation between ROG and MFD, H-ROG and HFD

It assumes importance to reflect upon the observation that the correlation coefficients between ROG centric results and MFD centric results are high. We must not forget that it might often be erroneous to conclude that processes that produce same (or similar) results are same; merely because the results from them are matching (in some significant confidence interval). Two completely different systemic dynamics might provide us with the same results (Please refer to **Materials S6** for explicit examples). Since we are trying to understand a system as complex as proteins, we should look beyond the values (correlation coefficients and confidence intervals) and attempt continuously to refine our methodologies that describe interior dynamics with more and more honest constructs. The detailed set of reasons, on why ROG-centric measures for description of protein interior might not be as honest as FD-centric measures was discussed in the introduction. Hence, although these correlations (in the sphere of mass and to some extent hydrophobicity) are high; the lights in which underlying processes (behind these correlation coefficients) looked at biological reality are starkly different.

At the same time, we notice certain unexpected correlations in **Table-5** and **Table-7**, which challenges our present-day understanding of protein interior. For example, we find a non-trivial correlation between PFD and HFD in all-α, α/β and (especially) α+β proteins. Does this imply a small yet significant dependence of dielectric constant on hydrophobicity content of a protein (and vice-versa)? And if so, why so? What is the uniqueness of structural features in all-β proteins that neither their mass nor hydrophobicity distribution show any dependence on their polarizability distributions? Similarly, in the context of **Table-7** entries, one might be prompted to attribute the least space-filling nature of MFD and HFD in α+β proteins (with respect to their number of atoms) to the separation between α-helices and (predominantly, anti-parallel) β-sheet domains. But is it solely because of that? Another possible interpretation of the same might stem from the averaged nature of measurable properties in proteins with multiple domains. But then, why doesn't the scaling of mass and hydrophobicity in α/β proteins (with respect to their number of atoms) suffer from the same? - All these are not some simple questions for which ready-made and general answers can be found either from existing knowledge base or from the framework of the present work, merely. However, carefully designed unbiased studies that attempt to link microscopic features of protein interior to its bulk properties of proteins, will hopefully provide us with the desired knowledge.

This little work presented here, wanted to construct a reliable and holistic platform that can study the latent symmetry of self-similar profiles of some important biophysical properties in proteins, simultaneously. It came up with some unexpected results. A set of thorough analysis and debates based on these findings, might help us in our ultimate pursuit to understand proteins better.

Significance of the present study, is multidimensional. It proposes new tool-set (hydrophobic ROG, HFD, PFD) and concept (‘untapped-hydrophobicity’) to probe protein interior. Such coherent scheme of quantification of relevant biophysical parameters, on one hand, brings to fore the fallacy associated with certain traditional ideas concerning the extent of protein packing and nature of stability in all-α proteins. On the other hand, it bolsters the ideas regarding importance of high hydrophobicity and low-polarizability to ensure protein stability. From a different perspective, the idea of ‘untapped-hydrophobicity’ raises new opportunities in the field of protein engineering, because it suggests that proteins can be designed to ensure maximal packing but such extreme packing schemes might not ensure an efficient functioning of the same protein. Nature, as it appears from the findings of this study, sacrifices certain degrees of packing in the protein interior so that precise geometric natures of shape topography (and hence, the surface characteristics) are maintained; which ultimately ensures the functionality of a protein.

The existence (and possible reason) of ‘untapped hydrophobicity’ in three major structural classes; a conspicuous absence of the same from all-α class of proteins; a statistical quantification (that does not rely on distinguishing interior atoms) of polarizability within proteins, and a suggestion that perhaps it is not hydrophobicity but extremely low polarizability - what ensures stability of all-α proteins; - were all glimpses of new information that the present study has presented the evergreen field of ‘protein structure’ with.

## Materials and Methods

### Materials

The only materials used in the present study were biological units of all the proteins with high resolution (<3.0 Å) X-ray crystal structures, derived from a database of 373 pairs of structurally aligned (87.17%) proteins, drawn from thermophilic and mesophilic organisms [85]. Since the thermophilic proteins are known to posses superior packing characteristics [86], this dataset was chosen to ensure the presence of entire spectrum structural features. Advantages (in terms of biological relevance) of working with biological unit of a protein is elucidated recently [87], hence we chose to work with it, rather than working with the asymmetric unit information about the same. Furthermore, since there were proteins with multiple types of SCOP domains, to ensure logical uniformity of the analysis, care was taken to filter out only the proteins where the SCOP domains were the same. Thus, strictly speaking, this study was carried out on the protein domains of biological units of structurally aligned thermophilic and mesophilic proteins.

### Algorithm

#### Part-1) : Calculation for ROG and Hydrophobic-ROG

Although it has been established in the introduction (and Discussion) section that the measure ROG is replete with limitations when it comes to describe proteins as proteins, - it is undoubtedly the most established measure to probe whatever the present work attempts to probe. Hence, we undertook the calculation of ROG (and extended the existing algorithms to propose a new measure, hydrophobic-ROG that attempts to quantify the hydrophobicity distribution within the protein). This exercise was important, to prove undeniably that FD centric measures do not miss out on any information that ROG centric measures can provide the users with; but alongside the (already known) ROG centric information, it provides us with many more information.

The coordinate of the Centre of Mass (CM) of a protein can be calculated with the formula :

**CM  =  ( ∑_i_ m_i_ P_i_ ) / (∑_i_ m_i_ )** , where m_i_ and P_i_ denote the mass and position of any i^th^ atom of the protein.

The Hydrophobic Centre (HC) of a protein can be calculated using the same formula, but by only substituting mass of an atom by (residue-specific) magnitudes of atomic hydrophobicity.

The Radius of Gyration (ROG) (of an uncomplexed-protein) can be calculated with the formula :

**ROG  =  square root of (ROG)^2^**

**(ROG)^2^  =  ( ∑_i_ m_i_ ∥P_i_ − CM∥^2^ ) / (∑_i_ m_i_ )**

The hydrophobic ROG can be calculated in exactly the same way, just by substituting CM with HC. The hydrophobic ROG suffers from all the drawbacks that ROG has, but has the advantage that it can be easily related to (easily, but incorrectly).

#### Calculation for MFD, HFD, PFD

For any biophysical property, say the mass distribution, the algorithm can be implemented by describing the distribution as : **M ∼ R^FD^** (**M** : total mass of all the atoms of the protein, **R** : a characteristic length scale). FD can be calculated for an individual protein by plotting M contained inside concentric spheres of radius R from the CM of the protein, on a log-log scale. The invariant portion of slope of such a graph implies a scale-invariant (self-similar) distribution of the property under consideration for the protein; and FD for that protein for that property assumes this magnitude. The parallel (in some particular case, nearly parallel) stretch of ordinate with varying magnitude of abscissa implies that distribution of the property (plotted in ordinate) at that particular scale of probing the system, in invariant. Hence the protein can be said to possess a self-similar distribution of the property under consideration in that scaling range. This is a simple yet general scheme to detect the (hidden) organizational scheme of a complex system (protein) that connects the atomic level properties with the statistical properties of it.

The HFD can be calculated in similar manner, by merely substituting atomic mass with (residue-specific) atomic hydrophobicity magnitudes. Calculation of PFD involved identification of centroid of every amino acid (rather than the Cα atom of the same); because the centroid can be considered as a better representative of the location of the amino acid within the protein.

## Supporting Information

Materials S1 Mass-Hydrophobicity-Polarizability Fractal Dimension values across four major SCOP classes (Results of Table-1 suit) with detailed break-up for the thermophilic proteins.
(0.09 MB PDF)

Materials S2 Mass-Hydrophobicity-Polarizability Fractal Dimension values across four major SCOP classes (Results of Table-1 suit) with detailed break-up for the mesophilic proteins.
(0.09 MB PDF)

Materials S3 Detailed break-up of the components of Figure-5.
(0.22 MB DOC)

Materials S4 Another Interpretation Of Untapped Hydrophobicity
(0.02 MB DOC)

Materials S5 Comparison With Helix-Coil Transition Theory
(0.03 MB DOC)

Materials S6 Explicit examples to illustrate differences FD-centric measures and ROG-centric examples; although the correlation-coefficients between them might be high.
(0.02 MB DOC)

Materials S7 Break-up of Table-3, depicting the thermophilic and mesophilic contributions to each of these classes towards all the analyzed correlations.
(0.05 MB DOC)
