# Supplementary material for: Revisiting the Myths of Protein Interior: Studying Proteins with Mass-Fractal Hydrophobicity-Fractal and Polarizability-Fractal Dimensions
Source: PLoS One. 2009 Oct 16;4(10):e7361. doi: 10.1371/journal.pone.0007361 (PMC2760208; doi:10.1371/journal.pone.0007361)
Supplement: Materials S1 — Mass-Hydrophobicity-Polarizability Fractal Dimension values across four major SCOP classes (Results of Table-1 suit) with detailed break-up for the thermophilic proteins. (0.09 MB PDF) [file pone.0007361.s001.pdf]

Mass-FD versus Hydrophobic-FD comparison for
4 major SCOP classes of Thermophilic proteins

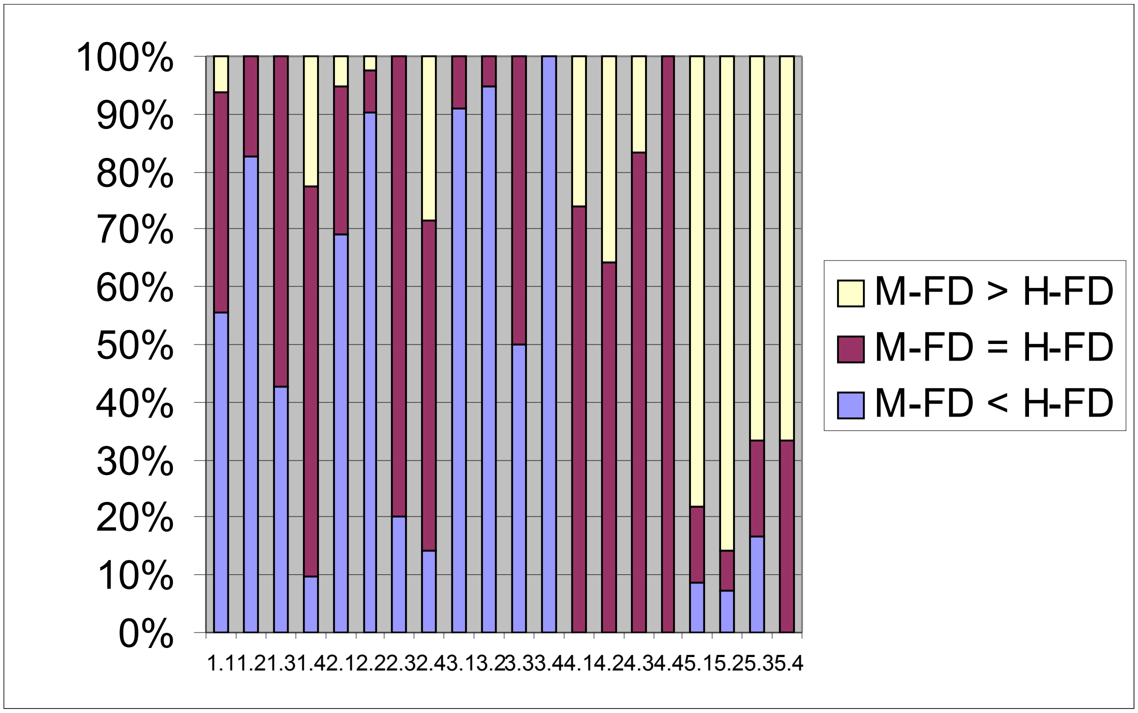

(Figure –1)

Legend :

1.1 : Entire α/β population of the thermophilic protein set
1.2 : Thermophilic α/β small proteins (set 1 (<5000 atoms))
1.3 : Thermophilic α/β moderately large proteins (set 2 (>5000 atoms) but (<8000 atoms))
1.4 : Thermophilic α/β large proteins (set 3 (>8000 atoms))

2.1 : Entire α+β population of the thermophilic protein set
2.2 : Thermophilic α+β small proteins (set 1 (<5000 atoms))
2.3 : Thermophilic α+β moderately large proteins (set 2 (>5000 atoms) but (<8000 atoms))
2.4 : Thermophilic α+β large proteins (set 3 (>8000 atoms))

3.1 : Entire All-β population of the thermophilic protein set
3.2 : Thermophilic All-β small proteins (set 1 (<5000 atoms))
3.3 : Thermophilic All-β moderately large proteins (set 2 (>5000 atoms) but (<8000 atoms))
3.4 : Thermophilic All-β large proteins (set 3 (>8000 atoms))

4.1 : Entire All-α population of the thermophilic protein set
4.2 : Thermophilic All-α small proteins (set 1 (<5000 atoms))
4.3 : Thermophilic All-α moderately large proteins (set 2 (>5000 atoms) but (<8000 atoms))
4.4 : Thermophilic All-α large proteins (set 3 (>8000 atoms))

5.1 : Entire All-α population of the thermophilic protein set
5.2 : Thermophilic All-α small proteins (set 1 (<5000 atoms))
5.3 : Thermophilic All-α moderately large proteins (set 2 (>5000 atoms) but (<8000 atoms))
5.4 : Thermophilic All-α large proteins (set 3 (>8000 atoms))
5.1, 5.2, 5.3, 5.4 – are all calculated with the consideration of 2nd decimal places.

TABULAR REPRESENTATION OF FIGURE – 1 FACTS

Protein Type	Mass-FD < Hydrophobic-FD	Mass-FD = Hydrophobic-FD	Mass-FD > Hydrophobic-FD
**Entire α/β**	**55.65%**	**38.2%**	**6.09%**
α/β(SET-1)	82.54%	17.46%	0%
α/β(SET-2)	42.86%	57.14%	0%
α/β(SET-3)	9.68%	67.74%	22.58%
**Entire α+β**	**68.97%**	**25.86%**	**5.17%**
α+β (SET-1)	90.24%	7.32%	2.44%
α+β (SET-2)	20.00%	80.00%	0%
α+β (SET-3)	14.29%	57.14%	28.57%
**Entire All-β**	**90.91%**	**9.09%**	**0%**
All-β (SET-1)	94.74%	5.26%	0%
All-β (SET-2)	50.00%	50.00%	0%
All-β (SET-3)	100.00%	0%	0%
**Entire All-α**	**0%**	**73.91%**	**26.09%**
All-α (SET-1)	0%	64.29%	35.71%
All-α (SET-2)	0%	83.33%	16.67%
All-α (SET-3)	0%	100%	0%
**Entire All-α With 2nd dec. place**	**8.70%**	**13.04%**	**78.26%**
**All-α (SET-1) With 2nd dec. place**	7.14%	7.14%	85.71%
**All-α (SET-2) With 2nd dec. place**	16.67%	16.67%	66.67%
**All-α (SET-3) With 2nd dec. place**	0%	33.33%	66.67%

Table -1

LEGEND (For Table-1)::

Extremo Mass-Vs-Hydroph Fd Comparison.

Bin-1 Represents Extrm-Alpha/Beta,
Bin-2 Represents Extrm-Alpha+Beta,
Bin-3 Represents Extrm-All-Beta,
Bin-4 Represents Extrm-All-Alpha,
Bin-5 Represents Extrm-All-Alpha (with 2nd decimal places).

Column – 1 Specification :
1.1, 2.1, 3.1, 4.1 - Represent Entire Protein Set Population For
Extrm-A/B, Extrm-A+B, Extrm-All-B, Extrm-All-A - Respectively.
5.1 - Represents Extrm-All-A With 2nd Decimal Places.

1.2, 2.2, 3.2, 4.2, 5.2
- Represent Set-1(<5000 Atom) Proteins For Respective Classes.
1.3, 2.3, 3.3, 4.3, 5.3
- Represent Set-2(>5000 Atom But <8000 Atoms) Proteins For Respective Classes.
1.4, 2.4, 3.4, 4.4, 5.4
- Represent Set-1(<5000 Atom) Proteins For Respective Classes.

Column 2 Describes M-Fd<H-Fd Population In Percentage
Column 3 Describes M-Fd=H-Fd Population In Percentage
Column 4 Describes M-Fd>H-Fd Population In Percentage
