# Supplementary material for: Revisiting the Myths of Protein Interior: Studying Proteins with Mass-Fractal Hydrophobicity-Fractal and Polarizability-Fractal Dimensions
Source: PLoS One. 2009 Oct 16;4(10):e7361. doi: 10.1371/journal.pone.0007361 (PMC2760208; doi:10.1371/journal.pone.0007361)
Supplement: Materials S2 — Mass-Hydrophobicity-Polarizability Fractal Dimension values across four major SCOP classes (Results of Table-1 suit) with detailed break-up for the mesophilic proteins. (0.09 MB PDF) [file pone.0007361.s002.pdf]

Mass-FD versus Hydrophobic-FD comparison for 4 major SCOP classes of Mesophilic proteins

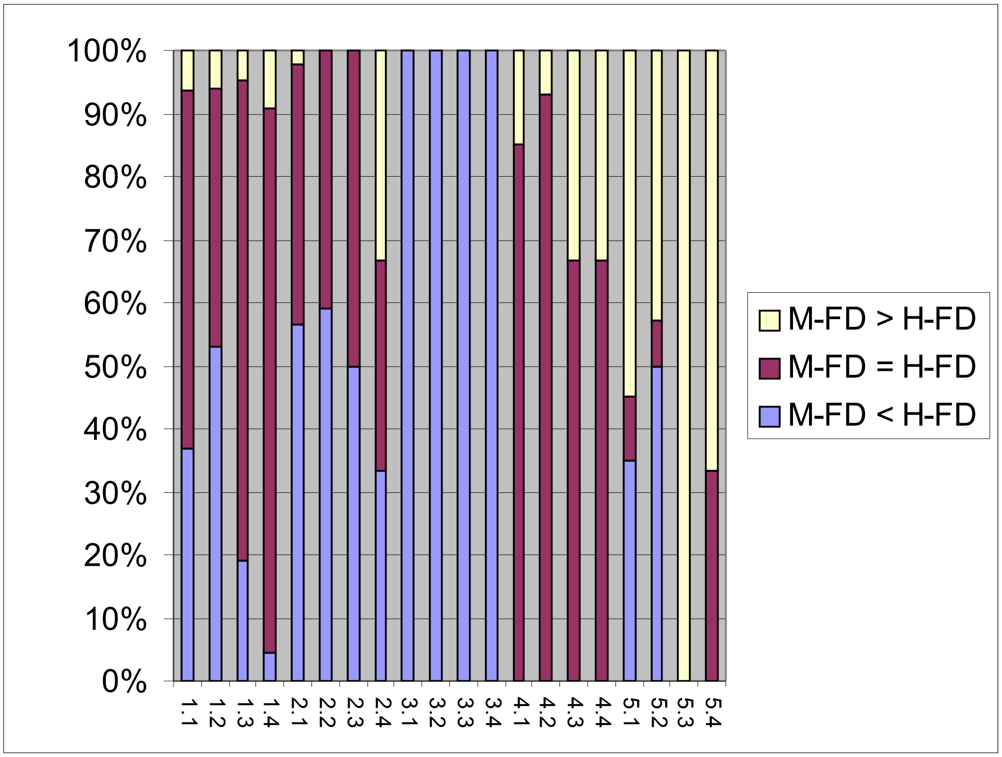

(Figure –1)

Legend :

1.1 : Entire α/β population of the mesophilic protein set
1.2 : Mesophilic α/β small proteins (set 1 (<5000 atoms))
1.3 : Mesophilic α/β moderately large proteins (set 2 (>5000 atoms) but (<8000 atoms))
1.4 : Mesophilic α/β large proteins (set 3 (>8000 atoms))

2.1 : Entire α+β population of the mesophilic protein set
2.2 : Mesophilic α+β small proteins (set 1 (<5000 atoms))
2.3 : Mesophilic α+β moderately large proteins (set 2 (>5000 atoms) but (<8000 atoms))
2.4 : Mesophilic α+β large proteins (set 3 (>8000 atoms))

3.1 : Entire All-β population of the mesophilic protein set
3.2 : Mesophilic All-β small proteins (set 1 (<5000 atoms))
3.3 : Mesophilic All-β moderately large proteins (set 2 (>5000 atoms) but (<8000 atoms))
3.4 : Mesophilic All-β large proteins (set 3 (>8000 atoms))

4.1 : Entire All-α population of the mesophilic protein set
4.2 : Mesophilic All-α small proteins (set 1 (<5000 atoms))
4.3 : Mesophilic All-α moderately large proteins (set 2 (>5000 atoms) but (<8000 atoms))
4.4 : Mesophilic All-α large proteins (set 3 (>8000 atoms))

5.1 : Entire All-α population of the mesophilic protein set
5.2 : Mesophilic All-α small proteins (set 1 (<5000 atoms))
5.3 : Mesophilic All-α moderately large proteins (set 2 (>5000 atoms) but (<8000 atoms))
5.4 : Mesophilic All-α large proteins (set 3 (>8000 atoms))
5.1, 5.2, 5.3, 5.4 – are all calculated with the consideration of 2nd decimal places.

TABULAR REPRESENTATION OF FIGURE – 1 FACTS

Protein Type	Mass-FD < Hydrophobic-FD	Mass-FD = Hydrophobic-FD	Mass-FD > Hydrophobic-FD
**Entire α/β**	**36.94%**	**56.76%**	**6.31%**
α/β(SET-1)	52.94%	41.18%	5.88%
α/β(SET-2)	19.05%	76.19%	4.76%
α/β(SET-3)	4.55%	86.36%	9.09%
**Entire α+β**	**56.52%**	**41.30%**	**2.17%**
α+β (SET-1)	58.97%	41.02%	0%
α+β (SET-2)	50.00%	50.00%	0%
α+β (SET-3)	33.33%	33.33%	33.33%
**Entire All-β**	**100%**	**0%**	**0%**
All-β (SET-1)	100%	0%	0%
All-β (SET-2)	100%	0%	0%
All-β (SET-3)	100%	0%	0%
**Entire All-α**	**0%**	**85%**	**15%**
All-α (SET-1)	0%	66.67%	33.33%
All-α (SET-2)	0%	66.67%	33.33%
All-α (SET-3)	0%	66.67%	33.33%
**Entire All-α With 2nd dec. place**	**35%**	**10%**	**55%**
**All-α (SET-1) With 2nd dec. place**	50%	7.14%	42.86%
**All-α (SET-2) With 2nd dec. place**	0%	0%	100%
**All-α (SET-3) With 2nd dec. place**	0%	33.33%	66.67%

Table -1

LEGEND (For Table-1)::

Meso Mass-Vs-Hydroph Fd Comparison.

Bin-1 Represents Meso-Alpha/Beta,
Bin-2 Represents Meso -Alpha+Beta,
Bin-3 Represents Meso -All-Beta,
Bin-4 Represents Meso -All-Alpha,
Bin-5 Represents Meso -All-Alpha (with 2nd decimal places).

Column – 1 Specification :
1.1, 2.1, 3.1, 4.1 - Represent Entire Protein Set Population For
Meso -A/B, Meso -A+B, Meso -All-B, Meso -All-A - Respectively.
5.1 - Represents Meso -All-A With 2nd Decimal Places.

1.2, 2.2, 3.2, 4.2, 5.2
- Represent Set-1(<5000 Atom) Proteins For Respective Classes.

1.3, 2.3, 3.3, 4.3, 5.3
- Represent Set-2(>5000 Atom But <8000 Atoms) Proteins For Respective Classes.

1.4, 2.4, 3.4, 4.4, 5.4
- Represent Set-1(<5000 Atom) Proteins For Respective Classes.

Column 2 Describes M-Fd<H-Fd Population In Percentage
Column 3 Describes M-Fd=H-Fd Population In Percentage
Column 4 Describes M-Fd>H-Fd Population In Percentage
