# Supplementary material for: Revisiting the Myths of Protein Interior: Studying Proteins with Mass-Fractal Hydrophobicity-Fractal and Polarizability-Fractal Dimensions
Source: PLoS One. 2009 Oct 16;4(10):e7361. doi: 10.1371/journal.pone.0007361 (PMC2760208; doi:10.1371/journal.pone.0007361)
Supplement: Materials S3 — Detailed break-up of the components of Figure-5. (0.22 MB DOC) [file pone.0007361.s003.doc]

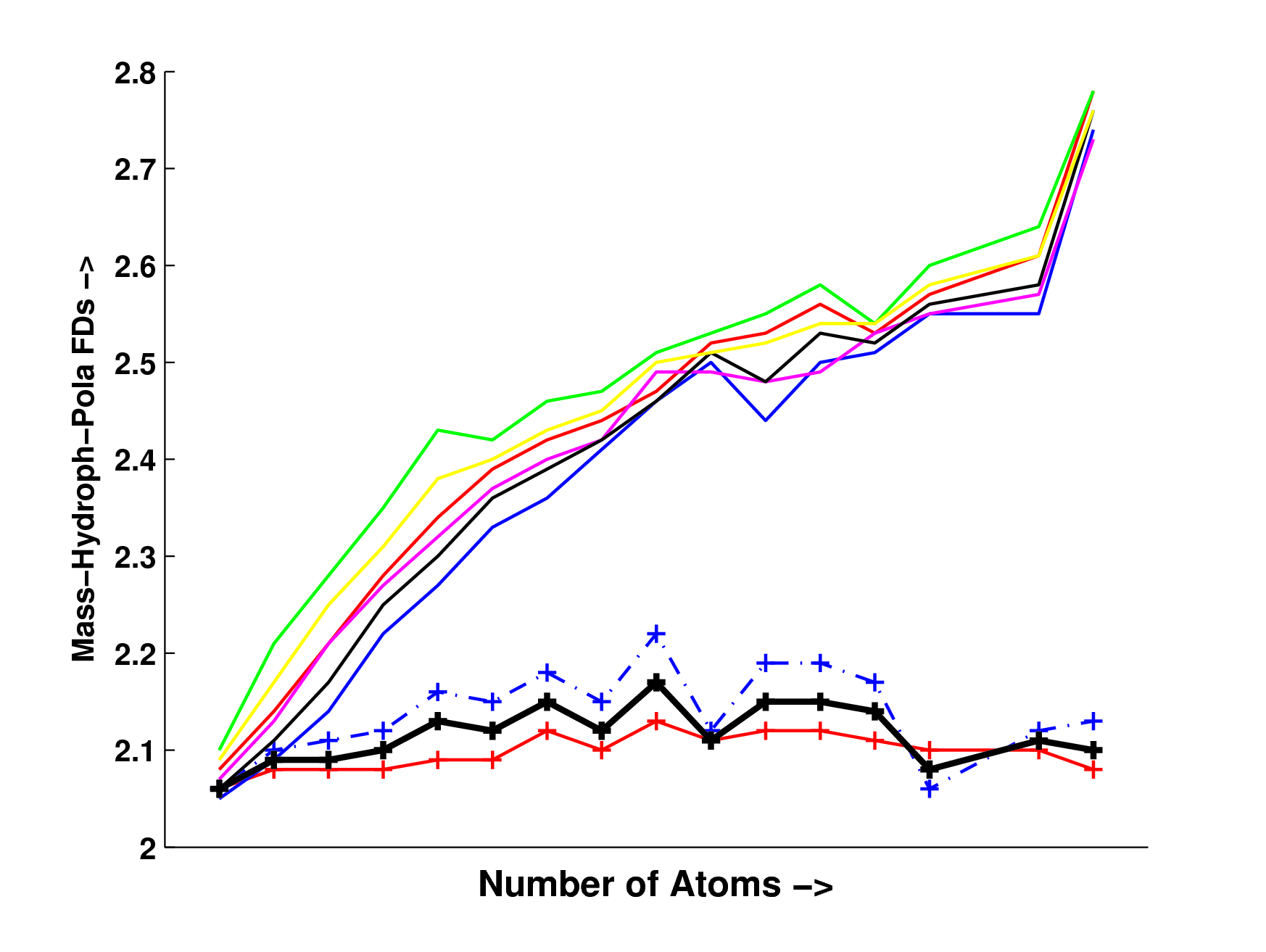


Figure – 5

Legends :

— : Distribution of Thermophilic Mass­FD

— : Distribution of Thermophilic Hydrophobic­FD

+—+ : Distribution of Thermophilic Polarizability­FD

— : Distribution of Mesophilic Mass­FD

— : Distribution of Mesophilic Hydrophobic­FD

+—+ : Distribution of Mesophilic Polarizability­FD

— : Distribution of Mass­FD of the entire protein set.

— : Distribution of Hydrophobic­FD of the entire protein set.

+—+ : Distribution of Polarizability­FD of the entire protein set.

ENTIRE ALGORITHM BEHIND FIGURE­5 PLOT

BIN THE EXTREMOPHILIC PROTEINS IN 17 BINS.

BIN 1 ­ (PROTEINS WITH LESS THAN OR EQUAL TO 1000 ATOMS).

BIN 2 ­ (PROTEINS WITH GREATER THAN 1000 BUT LESS THAN OR EQUAL TO 2000 ATOMS). BIN 3 ­ (PROTEINS WITH GREATER THAN 2000 BUT LESS THAN OR EQUAL TO 3000 ATOMS). BIN 4 ­ (PROTEINS WITH GREATER THAN 3000 BUT LESS THAN OR EQUAL TO 4000 ATOMS). BIN 5 ­ (PROTEINS WITH GREATER THAN 4000 BUT LESS THAN OR EQUAL TO 5000 ATOMS). BIN 6 ­ (PROTEINS WITH GREATER THAN 5000 BUT LESS THAN OR EQUAL TO 6000 ATOMS). BIN 7 ­ (PROTEINS WITH GREATER THAN 6000 BUT LESS THAN OR EQUAL TO 7000 ATOMS). BIN 8 ­ (PROTEINS WITH GREATER THAN 7000 BUT LESS THAN OR EQUAL TO 8000 ATOMS). BIN 9 ­ (PROTEINS WITH GREATER THAN 8000 BUT LESS THAN OR EQUAL TO 9000 ATOMS). BIN 10 ­ (PROTEINS WITH GREATER THAN 9000 BUT LESS THAN OR EQUAL TO 10000 ATOMS). BIN 11 ­ (PROTEINS WITH GREATER THAN 10000 BUT LESS THAN OR EQUAL TO 11000 ATOMS). BIN 12 ­ (PROTEINS WITH GREATER THAN 11000 BUT LESS THAN OR EQUAL TO 12000 ATOMS). BIN 13 ­ (PROTEINS WITH GREATER THAN 12000 BUT LESS THAN OR EQUAL TO 13000 ATOMS). BIN 14 ­ (PROTEINS WITH GREATER THAN 13000 BUT LESS THAN OR EQUAL TO 14000 ATOMS). BIN 15 ­ (PROTEINS WITH GREATER THAN 14000 BUT LESS THAN OR EQUAL TO 15000 ATOMS). BIN 16 ­ (PROTEINS WITH GREATER THAN 15000 BUT LESS THAN OR EQUAL TO 20000 ATOMS)

BIN 17 ­ (PROTEINS WITH GREATER THAN 20000 BUT LESS THAN OR EQUAL TO **MAX** ATOMS)

// **MAX** WOULD HAVE TO BE IDENTIFIED. MAX IS CALCULATED FOR THE ENTIRE DATASET, IRRESPECTIVE OF EXTREMO OR MESO.

CALCULATE THE MEAN M­FD FOR ECH OF THESE BINS.

PLOT THE M­FDS VERSUS #ATOMS GRAPH FOR EXTREMOPHILIC PROTEINS.

BIN THE MESOPHILIC PROTEINS IN 17 BINS.

BIN 1 ­ (PROTEINS WITH LESS THAN OR EQUAL TO 1000 ATOMS).

BIN 2 ­ (PROTEINS WITH GREATER THAN 1000 BUT LESS THAN OR EQUAL TO 2000 ATOMS). BIN 3 ­ (PROTEINS WITH GREATER THAN 2000 BUT LESS THAN OR EQUAL TO 3000 ATOMS). BIN 4 ­ (PROTEINS WITH GREATER THAN 3000 BUT LESS THAN OR EQUAL TO 4000 ATOMS). BIN 5 ­ (PROTEINS WITH GREATER THAN 4000 BUT LESS THAN OR EQUAL TO 5000 ATOMS). BIN 6 ­ (PROTEINS WITH GREATER THAN 5000 BUT LESS THAN OR EQUAL TO 6000 ATOMS). BIN 7 ­ (PROTEINS WITH GREATER THAN 6000 BUT LESS THAN OR EQUAL TO 7000 ATOMS). BIN 8 ­ (PROTEINS WITH GREATER THAN 7000 BUT LESS THAN OR EQUAL TO 8000 ATOMS). BIN 9 ­ (PROTEINS WITH GREATER THAN 8000 BUT LESS THAN OR EQUAL TO 9000 ATOMS). BIN 10 ­ (PROTEINS WITH GREATER THAN 9000 BUT LESS THAN OR EQUAL TO 10000 ATOMS). BIN 11 ­ (PROTEINS WITH GREATER THAN 10000 BUT LESS THAN OR EQUAL TO 11000 ATOMS). BIN 12 ­ (PROTEINS WITH GREATER THAN 11000 BUT LESS THAN OR EQUAL TO 12000 ATOMS). BIN 13 ­ (PROTEINS WITH GREATER THAN 12000 BUT LESS THAN OR EQUAL TO 13000 ATOMS). BIN 14 ­ (PROTEINS WITH GREATER THAN 13000 BUT LESS THAN OR EQUAL TO 14000 ATOMS). BIN 15 ­ (PROTEINS WITH GREATER THAN 14000 BUT LESS THAN OR EQUAL TO 15000 ATOMS).

// NO DATA FOUND FPR THIS BIN.

BIN 16 ­ (PROTEINS WITH GREATER THAN 15000 BUT LESS THAN OR EQUAL TO 20000 ATOMS)

BIN 17 ­ (PROTEINS WITH GREATER THAN 20000 BUT LESS THAN OR EQUAL TO **MAX** ATOMS)

// **MAX** WOULD HAVE TO BE IDENTIFIED. MAX IS CALCULATED FOR THE ENTIRE DATASET, IRRESPECTIVE OF EXTREMO OR MESO.

CALCULATE THE MEAN M­FD FOR ECH OF THESE BINS.

PLOT THE M­FD VERSUS #ATOMS GRAPH FOR MESOPHILIC PROTEINS.

RESULTS OBTAINED FROM IMPLEMENTATION OF THE ALGORITHM NARRATED ABOVE

DATA FOR EXTREMOPHILIC MASS­FD DISTRIBUTION (ON THE Y­AXIS) VERSUS #ATOMS ON THE X­AXIS PLOT :

EXTREMOPHILIC BIN­ 1) MASS­FD MEAN = 2.08 ­ THIS WILL BE PLOTTED ON Y AXIS, WITH 1000

BEING THE X AXIS VALUE.

EXTREMOPHILIC BIN­ 2) MASS­FD MEAN = 2.14 ­ THIS WILL BE PLOTTED ON Y AXIS, WITH 2000

BEING THE X AXIS VALUE.

EXTREMOPHILIC BIN­ 3) MASS­FD MEAN = 2.21 ­ THIS WILL BE PLOTTED ON Y AXIS, WITH 3000

BEING THE X AXIS VALUE.

EXTREMOPHILIC BIN­ 4) MASS­FD MEAN = 2.28 ­ THIS WILL BE PLOTTED ON Y AXIS, WITH 4000

BEING THE X AXIS VALUE.

EXTREMOPHILIC BIN­ 5) MASS­FD MEAN = 2.34 ­ THIS WILL BE PLOTTED ON Y AXIS, WITH 5000

BEING THE X AXIS VALUE.

EXTREMOPHILIC BIN­ 6) MASS­FD MEAN = 2.39 ­ THIS WILL BE PLOTTED ON Y AXIS, WITH 6000

BEING THE X AXIS VALUE.

EXTREMOPHILIC BIN­ 7) MASS­FD MEAN = 2.42 ­ THIS WILL BE PLOTTED ON Y AXIS, WITH 7000

BEING THE X AXIS VALUE.

EXTREMOPHILIC BIN­ 8) MASS­FD MEAN = 2.44 ­ THIS WILL BE PLOTTED ON Y AXIS, WITH 8000

BEING THE X AXIS VALUE.

EXTREMOPHILIC BIN­ 9) MASS­FD MEAN = 2.47 ­ THIS WILL BE PLOTTED ON Y AXIS, WITH 9000

BEING THE X AXIS VALUE.

EXTREMOPHILIC BIN­ 10) MASS­FD MEAN = 2.52 ­ THIS WILL BE PLOTTED ON Y AXIS, WITH 10000

BEING THE X AXIS VALUE.

EXTREMOPHILIC BIN­ 11) MASS­FD MEAN = 2.53 ­ THIS WILL BE PLOTTED ON Y AXIS, WITH 11000

BEING THE X AXIS VALUE.

EXTREMOPHILIC BIN­ 12) MASS­FD MEAN = 2.56 ­ THIS WILL BE PLOTTED ON Y AXIS, WITH 12000

BEING THE X AXIS VALUE.

EXTREMOPHILIC BIN­ 13) MASS­FD MEAN = 2.53 ­ THIS WILL BE PLOTTED ON Y AXIS, WITH 13000

BEING THE X AXIS VALUE.

EXTREMOPHILIC BIN­ 14) MASS­FD MEAN = 2.57 ­ THIS WILL BE PLOTTED ON Y AXIS, WITH 14000

BEING THE X AXIS VALUE.

EXTREMOPHILIC BIN­ 15) MASS­FD MEAN = NO DATA ­ THIS WILL BE PLOTTED ON Y AXIS, WITH

15000 BEING THE X AXIS VALUE. // NO DATA

EXTREMOPHILIC BIN­ 16) MASS­FD MEAN = 2.61 ­ THIS WILL BE PLOTTED ON Y AXIS, WITH 20000

BEING THE X AXIS VALUE.

EXTREMOPHILIC BIN­ 17) MASS­FD MEAN = 2.78 ­ THIS WILL BE PLOTTED ON Y AXIS, WITH GREATER THAN 20000 BEING THE X AXIS VALUE.

DATA FOR EXTREMOPHILIC HYDROPH­FD DISTRIBUTION (ON THE Y­AXIS) VERSUS #ATOMS ON THE X­AXIS PLOT :

EXTREMOPHILIC BIN­ 1) HYDROPH­FD MEAN = 2.10 ­ THIS WILL BE PLOTTED ON Y AXIS, WITH 1000

BEING THE X AXIS VALUE.

EXTREMOPHILIC BIN­ 2) HYDROPH­FD MEAN = 2.21 ­ THIS WILL BE PLOTTED ON Y AXIS, WITH 2000

BEING THE X AXIS VALUE.

EXTREMOPHILIC BIN­ 3) HYDROPH­FD MEAN = 2.28 ­ THIS WILL BE PLOTTED ON Y AXIS, WITH 3000

BEING THE X AXIS VALUE.

EXTREMOPHILIC BIN­ 4) HYDROPH­FD MEAN = 2.35 ­ THIS WILL BE PLOTTED ON Y AXIS, WITH 4000

BEING THE X AXIS VALUE.

EXTREMOPHILIC BIN­ 5) HYDROPH­FD MEAN = 2.43 ­ THIS WILL BE PLOTTED ON Y AXIS, WITH 5000

BEING THE X AXIS VALUE.

EXTREMOPHILIC BIN­ 6) HYDROPH­FD MEAN = 2.42 ­ THIS WILL BE PLOTTED ON Y AXIS, WITH 6000

BEING THE X AXIS VALUE.

EXTREMOPHILIC BIN­ 7) HYDROPH­FD MEAN = 2.46 ­ THIS WILL BE PLOTTED ON Y AXIS, WITH 7000

BEING THE X AXIS VALUE.

EXTREMOPHILIC BIN­ 8) HYDROPH­FD MEAN = 2.47 ­ THIS WILL BE PLOTTED ON Y AXIS, WITH 8000

BEING THE X AXIS VALUE.

EXTREMOPHILIC BIN­ 9) HYDROPH­FD MEAN = 2.51 ­ THIS WILL BE PLOTTED ON Y AXIS, WITH 9000

BEING THE X AXIS VALUE.

EXTREMOPHILIC BIN­ 10) HYDROPH­FD MEAN = 2.53 ­ THIS WILL BE PLOTTED ON Y AXIS, WITH 10000

BEING THE X AXIS VALUE.

EXTREMOPHILIC BIN­ 11) HYDROPH­FD MEAN = 2.55 ­ THIS WILL BE PLOTTED ON Y AXIS, WITH 11000

BEING THE X AXIS VALUE.

EXTREMOPHILIC BIN­ 12) HYDROPH­FD MEAN = 2.58 ­ THIS WILL BE PLOTTED ON Y AXIS, WITH 12000

BEING THE X AXIS VALUE.

EXTREMOPHILIC BIN­ 13) HYDROPH­FD MEAN = 2.54 ­ THIS WILL BE PLOTTED ON Y AXIS, WITH 13000

BEING THE X AXIS VALUE.

EXTREMOPHILIC BIN­ 14) HYDROPH­FD MEAN = 2.60 ­ THIS WILL BE PLOTTED ON Y AXIS, WITH 14000

BEING THE X AXIS VALUE.

EXTREMOPHILIC BIN­ 15) HYDROPH­FD MEAN = NO DATA­ THIS WILL BE PLOTTED ON Y AXIS, WITH

15000 BEING THE X AXIS VALUE. // NO DATA

EXTREMOPHILIC BIN­ 16) HYDROPH­FD MEAN = 2.64 ­ THIS WILL BE PLOTTED ON Y AXIS, WITH 20000

BEING THE X AXIS VALUE.

EXTREMOPHILIC BIN­ 17) HYDROPH­FD MEAN = 2.78 ­ THIS WILL BE PLOTTED ON Y AXIS, WITH GREATER THAN 20000 BEING THE X AXIS VALUE.

DATA FOR EXTREMOPHILIC POLARIZABILITY­FD DISTRIBUTION (ON THE Y­AXIS) VERSUS #ATOMS ON THE X­AXIS PLOT :

EXTREMOPHILIC BIN­ 1) POLA­FD MEAN = 2.06 ­ THIS WILL BE PLOTTED ON Y AXIS, WITH 1000

BEING THE X AXIS VALUE.

EXTREMOPHILIC BIN­ 2) POLA­FD MEAN = 2.08 ­ THIS WILL BE PLOTTED ON Y AXIS, WITH 2000

BEING THE X AXIS VALUE.

EXTREMOPHILIC BIN­ 3) POLA­FD MEAN = 2.08 ­ THIS WILL BE PLOTTED ON Y AXIS, WITH 3000

BEING THE X AXIS VALUE.

EXTREMOPHILIC BIN­ 4) POLA­FD MEAN = 2.08 ­ THIS WILL BE PLOTTED ON Y AXIS, WITH 4000

BEING THE X AXIS VALUE.

EXTREMOPHILIC BIN­ 5) POLA­FD MEAN = 2.09 ­ THIS WILL BE PLOTTED ON Y AXIS, WITH 5000

BEING THE X AXIS VALUE.

EXTREMOPHILIC BIN­ 6) POLA­FD MEAN = 2.09 ­ THIS WILL BE PLOTTED ON Y AXIS, WITH 6000

BEING THE X AXIS VALUE.

EXTREMOPHILIC BIN­ 7) POLA­FD MEAN = 2.12 ­ THIS WILL BE PLOTTED ON Y AXIS, WITH 7000

BEING THE X AXIS VALUE.

EXTREMOPHILIC BIN­ 8) POLA­FD MEAN = 2.10 ­ THIS WILL BE PLOTTED ON Y AXIS, WITH 8000

BEING THE X AXIS VALUE.

EXTREMOPHILIC BIN­ 9) POLA­FD MEAN = 2.13 ­ THIS WILL BE PLOTTED ON Y AXIS, WITH 9000

BEING THE X AXIS VALUE.

EXTREMOPHILIC BIN­ 10) POLA­FD MEAN = 2.11 ­ THIS WILL BE PLOTTED ON Y AXIS, WITH 10000

BEING THE X AXIS VALUE.

EXTREMOPHILIC BIN­ 11) POLA­FD MEAN = 2.12 ­ THIS WILL BE PLOTTED ON Y AXIS, WITH 11000

BEING THE X AXIS VALUE.

EXTREMOPHILIC BIN­ 12) POLA­FD MEAN = 2.12 ­ THIS WILL BE PLOTTED ON Y AXIS, WITH 12000

BEING THE X AXIS VALUE.

EXTREMOPHILIC BIN­ 13) POLA­FD MEAN = 2.11 ­ THIS WILL BE PLOTTED ON Y AXIS, WITH 13000

BEING THE X AXIS VALUE.

EXTREMOPHILIC BIN­ 14) POLA­FD MEAN = 2.10 ­ THIS WILL BE PLOTTED ON Y AXIS, WITH 14000

BEING THE X AXIS VALUE.

EXTREMOPHILIC BIN­ 15) POLA­FD MEAN = NO DATA ­ THIS WILL BE PLOTTED ON Y AXIS, WITH

15000 BEING THE X AXIS VALUE. // NO DATA

EXTREMOPHILIC BIN­ 16) POLA­FD MEAN = 2.10 ­ THIS WILL BE PLOTTED ON Y AXIS, WITH 20000

BEING THE X AXIS VALUE.

EXTREMOPHILIC BIN­ 17) POLA­FD MEAN = 2.08 ­ THIS WILL BE PLOTTED ON Y AXIS, WITH GREATER THAN 20000 BEING THE X AXIS VALUE.

DATA FOR MESOPHILIC MASS­FD DISTRIBUTION (ON THE Y­AXIS) VERSUS #ATOMS ON THE X­AXIS PLOT :

MESOPHILIC BIN­ 1) MASS­FD MEAN = 2.05 ­ THIS WILL BE PLOTTED ON Y AXIS, WITH 1000 BEING THE X AXIS VALUE.

MESOPHILIC BIN­ 2) MASS­FD MEAN = 2.09 ­ THIS WILL BE PLOTTED ON Y AXIS, WITH 2000 BEING THE

X AXIS VALUE.

MESOPHILIC BIN­ 3) MASS­FD MEAN = 2.14 ­ THIS WILL BE PLOTTED ON Y AXIS, WITH 3000 BEING THE X AXIS VALUE.

MESOPHILIC BIN­ 4) MASS­FD MEAN = 2.22 ­ THIS WILL BE PLOTTED ON Y AXIS, WITH 4000 BEING THE

X AXIS VALUE.

MESOPHILIC BIN­ 5) MASS­FD MEAN = 2.27 ­ THIS WILL BE PLOTTED ON Y AXIS, WITH 5000 BEING THE X AXIS VALUE.

MESOPHILIC BIN­ 6) MASS­FD MEAN = 2.33 ­ THIS WILL BE PLOTTED ON Y AXIS, WITH 6000 BEING THE

X AXIS VALUE.

MESOPHILIC BIN­ 7) MASS­FD MEAN = 2.36 ­ THIS WILL BE PLOTTED ON Y AXIS, WITH 7000 BEING THE X AXIS VALUE.

MESOPHILIC BIN­ 8) MASS­FD MEAN = 2.41 ­ THIS WILL BE PLOTTED ON Y AXIS, WITH 8000 BEING THE

X AXIS VALUE.

MESOPHILIC BIN­ 9) MASS­FD MEAN = 2.46 ­ THIS WILL BE PLOTTED ON Y AXIS, WITH 9000 BEING THE X AXIS VALUE.

MESOPHILIC BIN­ 10) MASS­FD MEAN = 2.50 ­ THIS WILL BE PLOTTED ON Y AXIS, WITH 10000 BEING THE

X AXIS VALUE.

MESOPHILIC BIN­ 11) MASS­FD MEAN = 2.44 ­ THIS WILL BE PLOTTED ON Y AXIS, WITH 11000 BEING THE X AXIS VALUE.

MESOPHILIC BIN­ 12) MASS­FD MEAN = 2.50 ­ THIS WILL BE PLOTTED ON Y AXIS, WITH 12000 BEING THE

X AXIS VALUE.

MESOPHILIC BIN­ 13) MASS­FD MEAN = 2.51 ­ THIS WILL BE PLOTTED ON Y AXIS, WITH 13000 BEING THE X AXIS VALUE.

MESOPHILIC BIN­ 14) MASS­FD MEAN = 2.55 ­ THIS WILL BE PLOTTED ON Y AXIS, WITH 14000 BEING THE

X AXIS VALUE.

MESOPHILIC BIN­ 15) MASS­FD MEAN = 2.51 ­ THIS WILL BE PLOTTED ON Y AXIS, WITH 15000 BEING THE X AXIS VALUE. // NO DATA

MESOPHILIC BIN­ 16) MASS­FD MEAN = 2.55 ­ THIS WILL BE PLOTTED ON Y AXIS, WITH 20000 BEING THE

X AXIS VALUE.

MESOPHILIC BIN­ 17) MASS­FD MEAN = 2.74 ­ THIS WILL BE PLOTTED ON Y AXIS, WITH GREATER THAN

20000 BEING THE X AXIS VALUE.

DATA FOR MESOPHILIC HYDROPH­FD DISTRIBUTION (ON THE Y­AXIS) VERSUS #ATOMS ON THE X­AXIS PLOT :

MESOPHILIC BIN­ 1) HYDROPH­FD MEAN = 2.07 ­ THIS WILL BE PLOTTED ON Y AXIS, WITH 1000

BEING THE X AXIS VALUE.

MESOPHILIC BIN­ 2) HYDROPH­FD MEAN = 2.13 ­ THIS WILL BE PLOTTED ON Y AXIS, WITH 2000

BEING THE X AXIS VALUE.

MESOPHILIC BIN­ 3) HYDROPH­FD MEAN = 2.21 ­ THIS WILL BE PLOTTED ON Y AXIS, WITH 3000

BEING THE X AXIS VALUE.

MESOPHILIC BIN­ 4) HYDROPH­FD MEAN = 2.27 ­ THIS WILL BE PLOTTED ON Y AXIS, WITH 4000

BEING THE X AXIS VALUE.

MESOPHILIC BIN­ 5) HYDROPH­FD MEAN = 2.32 ­ THIS WILL BE PLOTTED ON Y AXIS, WITH 5000

BEING THE X AXIS VALUE.

MESOPHILIC BIN­ 6) HYDROPH­FD MEAN = 2.37 ­ THIS WILL BE PLOTTED ON Y AXIS, WITH 6000

BEING THE X AXIS VALUE.

MESOPHILIC BIN­ 7) HYDROPH­FD MEAN = 2.40 ­ THIS WILL BE PLOTTED ON Y AXIS, WITH 7000

BEING THE X AXIS VALUE.

MESOPHILIC BIN­ 8) HYDROPH­FD MEAN = 2.42 ­ THIS WILL BE PLOTTED ON Y AXIS, WITH 8000

BEING THE X AXIS VALUE.

MESOPHILIC BIN­ 9) HYDROPH­FD MEAN = 2.49 ­ THIS WILL BE PLOTTED ON Y AXIS, WITH 9000

BEING THE X AXIS VALUE.

MESOPHILIC BIN­ 10) HYDROPH­FD MEAN = 2.49 ­ THIS WILL BE PLOTTED ON Y AXIS, WITH 10000

BEING THE X AXIS VALUE.

MESOPHILIC BIN­ 11) HYDROPH­FD MEAN = 2.48 ­ THIS WILL BE PLOTTED ON Y AXIS, WITH 11000

BEING THE X AXIS VALUE.

MESOPHILIC BIN­ 12) HYDROPH­FD MEAN = 2.49 ­ THIS WILL BE PLOTTED ON Y AXIS, WITH 12000

BEING THE X AXIS VALUE.

MESOPHILIC BIN­ 13) HYDROPH­FD MEAN = 2.53 ­ THIS WILL BE PLOTTED ON Y AXIS, WITH 13000

BEING THE X AXIS VALUE.

MESOPHILIC BIN­ 14) HYDROPH­FD MEAN = 2.55 ­ THIS WILL BE PLOTTED ON Y AXIS, WITH 14000

BEING THE X AXIS VALUE.

MESOPHILIC BIN­ 15) HYDROPH­FD MEAN = 2.58 ­ THIS WILL BE PLOTTED ON Y AXIS, WITH 15000

BEING THE X AXIS VALUE. // NO DATA

MESOPHILIC BIN­ 16) HYDROPH­FD MEAN = 2.57 ­ THIS WILL BE PLOTTED ON Y AXIS, WITH 20000

BEING THE X AXIS VALUE.

MESOPHILIC BIN­ 17) HYDROPH­FD MEAN = 2.73 ­ THIS WILL BE PLOTTED ON Y AXIS, WITH GREATER THAN 20000 BEING THE X AXIS VALUE.

DATA FOR MESOPHILIC POLARIZABILITY­FD DISTRIBUTION (ON THE Y­AXIS) VERSUS #ATOMS ON THE X­AXIS PLOT :

MESOPHILIC BIN­ 1) POLA­FD MEAN = 2.06 ­ THIS WILL BE PLOTTED ON Y AXIS, WITH 1000 BEING THE X AXIS VALUE.

MESOPHILIC BIN­ 2) POLA­FD MEAN = 2.10 ­ THIS WILL BE PLOTTED ON Y AXIS, WITH 2000 BEING THE X AXIS VALUE.

MESOPHILIC BIN­ 3) POLA­FD MEAN = 2.11 ­ THIS WILL BE PLOTTED ON Y AXIS, WITH 3000 BEING THE X AXIS VALUE.

MESOPHILIC BIN­ 4) POLA­FD MEAN = 2.12 ­ THIS WILL BE PLOTTED ON Y AXIS, WITH 4000 BEING THE X AXIS VALUE.

MESOPHILIC BIN­ 5) POLA­FD MEAN = 2.16 ­ THIS WILL BE PLOTTED ON Y AXIS, WITH 5000 BEING THE X AXIS VALUE.

MESOPHILIC BIN­ 6) POLA­FD MEAN = 2.15 ­ THIS WILL BE PLOTTED ON Y AXIS, WITH 6000 BEING THE X AXIS VALUE.

MESOPHILIC BIN­ 7) POLA­FD MEAN = 2.18 ­ THIS WILL BE PLOTTED ON Y AXIS, WITH 7000 BEING THE X AXIS VALUE.

MESOPHILIC BIN­ 8) POLA­FD MEAN = 2.15 ­ THIS WILL BE PLOTTED ON Y AXIS, WITH 8000 BEING THE X AXIS VALUE.

MESOPHILIC BIN­ 9) POLA­FD MEAN = 2.22 ­ THIS WILL BE PLOTTED ON Y AXIS, WITH 9000 BEING THE X AXIS VALUE.

MESOPHILIC BIN­ 10) POLA­FD MEAN = 2.12 ­ THIS WILL BE PLOTTED ON Y AXIS, WITH 10000 BEING THE X AXIS VALUE.

MESOPHILIC BIN­ 11) POLA­FD MEAN = 2.19 ­ THIS WILL BE PLOTTED ON Y AXIS, WITH 11000 BEING THE X AXIS VALUE.

MESOPHILIC BIN­ 12) POLA­FD MEAN = 2.19 ­ THIS WILL BE PLOTTED ON Y AXIS, WITH 12000 BEING THE X AXIS VALUE.

MESOPHILIC BIN­ 13) POLA­FD MEAN = 2.17 ­ THIS WILL BE PLOTTED ON Y AXIS, WITH 13000 BEING THE X AXIS VALUE.

MESOPHILIC BIN­ 14) POLA­FD MEAN = 2.06 ­ THIS WILL BE PLOTTED ON Y AXIS, WITH 14000 BEING THE X AXIS VALUE.

MESOPHILIC BIN­ 15) POLA­FD MEAN = 2.13 ­ THIS WILL BE PLOTTED ON Y AXIS, WITH 15000 BEING THE X AXIS VALUE. // NO DATA

MESOPHILIC BIN­ 16) POLA­FD MEAN = 2.12 ­ THIS WILL BE PLOTTED ON Y AXIS, WITH 20000 BEING THE X AXIS VALUE.

MESOPHILIC BIN­ 17) POLA­FD MEAN = 2.13 ­ THIS WILL BE PLOTTED ON Y AXIS, WITH GREATER THAN

20000 BEING THE X AXIS VALUE.
