# Supplementary material for: Revisiting the Myths of Protein Interior: Studying Proteins with Mass-Fractal Hydrophobicity-Fractal and Polarizability-Fractal Dimensions
Source: PLoS One. 2009 Oct 16;4(10):e7361. doi: 10.1371/journal.pone.0007361 (PMC2760208; doi:10.1371/journal.pone.0007361)
Supplement: Materials S4 — Another Interpretation Of Untapped Hydrophobicity (0.02 MB DOC) [file pone.0007361.s004.doc]

# Supplementary Material - 4

# Relating 'untapped hydrophobicity' to

**Contemporary knowledge of protein structure**

Another interpretation to account for the existence of [+(HFD-MFD)] can be constructed from suitably sampling the enormous knowledge base generated by prevailing myths of describing protein interior. For example, while utmost compactness of protein core has been reported by several studies over the last thirty-five years[1,2]; another steady stream of studies, which report that packing of atoms on protein surface as loose, are making rounds over the last twenty-five years [3,4]. This is (obviously) expected, because surface atoms involve themselves in highly directional hydrogen bonding interactions, "trading-off" their closely-packed disposition of the protein core [5]. As a result of these, the net magnitude of compactness of the protein reduces and MFD assumes a less magnitude than corresponding HFD and therefore [+(HFD-MFD)]'s implication of untapped hydrophobicity can be considered impertinent. But, such framework of logic will be naive and wrong on two counts.

It is wrong, because it fails to notice the fact that magnitude of HFD, will suffer too on the protein surface and probabilities are high that magnitude of HFD will decrease more dramatically on the surface than the corresponding MFD. In the best possible scenario, MFD will lose out proportionately as the HFD. But that fails to account for the existence of positive magnitude of (HFD-MFD). Furthermore, the stream of logic narrated in the last paragraph can easily be branded as naive; because, going by that school of thought, for any work on the compactness of a protein, it will be imperative to mathematically describe its shapes (or find some clever way to avoid it without losing any biological information), and then mention precisely which atoms belonging to which residue within which radial threshold from the center of mass of that protein are counted under the banner "core of the protein"; before carrying on with the same methodology for all the possible radial thresholds for that protein; to finally offer us a spectrum of compactness values for the protein. - Although rigorous, such scheme to measure interior compactness might not be easy to accomplish. Nevertheless, such 'not-so-easy' profiling of protein interior has been carried out recently [6], but that couldn't provide us a single objective measure for compactness of biophysical properties. Hence, interpretation of [+(HFD-MFD)] as 'untapped hydrophobicity' appears to be the best with our contemporary knowledge of protein structures.

(References are kept on the next page).

**References :**

**1. Richards FM (1974) The interpretation of protein structures: total volume, group volume distributions and packing density. J Mol Biol 82 : 1–14**

**2. Harpaz Y, Gerstein M, Chothia C (1994) Volume Changes on Protein Folding. Structure 2 : 641-649.**

**3. Franks F (1983) Water. R. Soc. Chem, London.**

**4. Gerstein M, Tsai J, Levitt M (1995) The volume of atoms on the protein surface: calculated from simulation, using Voronoi polyhedra. J Mol Biol 249 : 955–966.**

**5. Levitt M, Gerstein M, Huang E, Subbiah S, Tsai J (1997) Protein Folding: The Endgame; Ann Rev Biochem 66 : 549–579.**

**6. Banerji A, Ghosh I (2009) A new computational model to study mass inhomogeneity and hydrophobicity inhomogeneity in proteins; Eur. Biophys J 38 : 577-587.**
