# Supplementary material for: Revisiting the Myths of Protein Interior: Studying Proteins with Mass-Fractal Hydrophobicity-Fractal and Polarizability-Fractal Dimensions
Source: PLoS One. 2009 Oct 16;4(10):e7361. doi: 10.1371/journal.pone.0007361 (PMC2760208; doi:10.1371/journal.pone.0007361)
Supplement: Materials S5 — Comparison With Helix-Coil Transition Theory (0.03 MB DOC) [file pone.0007361.s005.doc]

# Supplementary Material - 5

# Comparison of the present theory with Helix-Coil Transition Theory :

To assess the justification and utility of the present model, it was crucial for us to compare it with an extremely popular model that describes helix-electrostatics and helix folding; namely, the Helix/Coil Transition Theory (HCTT). This comparison was of paramount interest because HCTT, as postulated in 1959 [1], had attempted to describe the energetics of systems formed by short polypeptide chains from a thermodynamic perspective and was successful to lay the foundation for algorithms to quantify possible helical tendencies in amino acid sequences. However, the original framework of Zimm and Bragg (rooted firmly in statistical mechanics) was later replaced by individual atomistic description of electrostatics of dipole formation (reviewed in [2,3]). Perhaps from this moment (about 35 years after Zimm-Bragg’s model), an electrostatic description of alpha-helix (stability and origin) started to dominate over the thermodynamic approach to describe the same. From this point onwards, although many a studies had attempted to relate the theoretical predictions to experimental data ([4,5] and references therein); various inconsistencies regarding the details of energetics of HCTT persisted. In a classic case of bottom-up (distinguishable, atomistic) versus top-down (indistinguishable, thermodynamic) approach; it was reported that electrostatic interactions are partially treated in HCTT [6,7]. (Only (i,i+1), (i,i+2), (i,i+3) and (i,i+4) interactions are considered, while charges separated by more than 20Å in a helical peptide can contribute to helix stability [6]). Furthermore, it is now established the HCTT cannot account for recently identified patterns in sequence motifs ("hydrophobic staple"([2,8]), "Pro-capping motif"[9], "Schellman motif"[10]) - that stabilize the helical conformation. Thus, although the HCTT had provided the basic template to examine the organizational features of alpha-helical stability; we know now, some 50 years down the line, that perhaps it is not capable of describing the enormous complexities present in helix architecture (either thermodynamically or electrostatically).

The algorithm presented here, on the other hand, operates on a different philosophy altogether; where the possibilities of such inconsistencies do not arise at the first place. By modeling the interior polarizability, electrostatic environment within a protein could be described from an uncomplicated yet most fundamental of point of views. Targeting this central property, viz. polarizability, we could ensure that our approach does not suffer from "the non-constancy in the magnitude of dielectric constant within the proteins" [11]. (Besides reliability, the proposed construct (PFD) is far more easily computable than another bottom-up marker to describe protein electrostatics, the "average dipole orientation"[12, 13]). Strength of electrostatic interactions in α-helices, α-helical dipoles and small all-α class of proteins has been studied previously by many [6,14,15]. These approaches, in general, were banking upon the origin and characterization of helical macro-dipoles, resulting from dipolar interactions between particular atoms belonging to particular amino acids. Protein atoms, viewed from this approach, were considered as distinguishable entities and not as indistinguishable ones, necessary for the statistical description of the system. Our approach of quantifying the conducive electrostatic environment within any all-α proteins is essentially statistical description of protein electrostatics; and hence it differs on crucial grounds from the aforementioned schools of thought.

**References :**

**1. Zimm BH, Bragg JK (1959). Theory of the phase transition between helix and random-coil. J Chem Phys 31 : 526-535.**

**2. Munoz V, Serrano L (1995) Helix design, prediction and stability. Curr Opin Biotech 6, 382-386.**

**3. Chakrabartty A, Baldwin RL (1995) Stability of α-helices. Adv Prot Chem 46 : 141-176.**

**4. Lomize AL, Mosberg HI (1997) Thermodynamic model of secondary structure for α-helical peptides and proteins. Biopolymers 42 : 239-269.**

**5. Andersen NH, Tong H (1998) Empirical parameterization of a model for predicting peptide helix/coil equilibrium populations. Prot Sci 6 : 1920-1936.**

**6. Lacroix E, Viguera AR, Serrano L (1998) Elucidating the Folding Problem of α-helices: local motifs, long-range electrostatics, ionic-strength dependence and prediction of NMR parameters; J Mol Biol 284 : 173-191.**

**7. Sitkof D, Lockhart DJ, Sharp KA, Honig AB (1994) Calculation of electrostatic effects at the amino terminus of an α-helix. Biophys J 67 : 2251-2260.**

**8. Creamer TP, Rose GD (1995) Interactions between hydrophobic side-chains within α-helices. Prot Sci 4 : 1305-1314.**

**9. Prieto J, Serrano L (1997) C-capping and helix stability: the Pro C-capping motif. J Mol Biol 274 : 276-288.**

**10. Viguera AR, Serrano L (1995) Experimental analysis of the Schellman motif. J Mol Biol.251 : 150-160.**

**11. Talley K, Ng C, Shoppell M, Kundrotas P, Alexov E (2008) On the electrostatic component of protein-protein binding free energy. PMC Biophysics 1:2.**

**12. Bastug T, Kuyucaka S (2007) Free energy simulations of single and double ion occupancy in gramicidin A. J Chem Phys 126 : 105103.**

**13. Saiz L, Klein ML (2005) The transmembrane domain of the acetylcholine receptor: insights from simulations on synthetic peptide models. Biophys J 88 : 959-970.**

**14. Huyghues-Despointes BMP, Baldwin RL (1997) Ion-pair and charged hydrogen-bond interactions between histidine and aspartate in a peptide helix. Biochemistry 36 : 1965-1970.**

15. Cochran DAE, Doig AJ (2001) Effect of the N2 residue on the stability of the α-helix for all 20 amino acids. Prot Sci 10 : 1305–1311.
