# Supplementary material for: Revisiting the Myths of Protein Interior: Studying Proteins with Mass-Fractal Hydrophobicity-Fractal and Polarizability-Fractal Dimensions
Source: PLoS One. 2009 Oct 16;4(10):e7361. doi: 10.1371/journal.pone.0007361 (PMC2760208; doi:10.1371/journal.pone.0007361)
Supplement: Materials S6 — Explicit examples to illustrate differences FD-centric measures and ROG-centric examples; although the correlation-coefficients between them might be high. (0.02 MB DOC) [file pone.0007361.s006.doc]

# Supplementary Material-6

**A note on the difference between ROG­centric and FD­centric values,**

**given that they have high correlation coefficients amongst them.**

It assumes importance to reflect upon the observation that the correlation coefficients between ROG centric results and MFD centric results are high.

We must not forget that we are students of science (and not accountants); therefore, mere values by themselves might not provide us with information about the systemic dynamics that produce these values. In fact, it might often be erroneous to conclude that processes that produce the same (or nearly the same) results are same; merely because the results are matching in some significant confidence interval. (Take two examples. First : ROG of a bowl of thick corn flex in milk with banana etc., might be the same as that of a blob of wet­soil. Although the ROG is same, systems are utterly different in their physical and chemical nature. Second : some person X shreds N ml of teardrops upon hearing the news

that he is blessed with a baby. That same person X shreds N ml of teardrops upon hearing the news that his mother is no more. ­ Obviously, systemic­dynamics underneath the process that produce the same "value"(viz. N ml) is different).

­ Thus two completely different systemic dynamics might provide us with the same results.

Coming back to the present context, we must start our study by acknowledging that proteins are not rock­solid objects, their shape is neither ellipsoidal nor spherical; ­ they are proteins, they look like proteins. Since we are trying to understand a system as complex as proteins, we should look beyond the values (correlation coefficients and confidence intervals) and attempt continuously to refine our methodologies that describe interior dynamics with more and more honest constructs. In that process, new information

might trickle in slowly; but hasn't it always been like that? (In other words, 1905 doesn't occur every year in the history of science). This little work wanted to construct an honest, reliable and holistic platform that can study the self­similar profiles of some important biophysical properties in proteins. It came up with some unexpected results. A set of thorough analysis and debates based on these results might help us in our ultimate pursuit to understand proteins better.
