# Supplementary material for: Revisiting the Myths of Protein Interior: Studying Proteins with Mass-Fractal Hydrophobicity-Fractal and Polarizability-Fractal Dimensions
Source: PLoS One. 2009 Oct 16;4(10):e7361. doi: 10.1371/journal.pone.0007361 (PMC2760208; doi:10.1371/journal.pone.0007361)
Supplement: Materials S7 — Break-up of Table-3, depicting the thermophilic and mesophilic contributions to each of these classes towards all the analyzed correlations. (0.05 MB DOC) [file pone.0007361.s007.doc]

**Supplementary Material-S7**

**Correlations amongst No. of atoms, Radius Of Gyration(ROG), MFD, Hydrophobic-ROG and HFD across major SCOP classes, segregated in Thermophilic and Mesophilic families.**

| **Correlations across SCOP classes** | **No. of atoms and MFD** | **No. of atoms and ROG** | **MFD and ROG** | **No. of atoms and HFD** | **No. of atoms and Hydrophobic-ROG** | **HFD and Hydrophobic-ROG** |
| --- | --- | --- | --- | --- | --- | --- |
| **Thermophilic**  **α/β** | **0.90** | **0.88** | **0.94** | **0.88** | **0.87** | **0.92** |
| **Mesophilic**  **α/β** | **0.88** | **0.92** | **0.92** | **0.88** | **0.92** | **0.92** |
| **Thermophilic**  **α+β** | **0.95** | **0.90** | **0.91** | **0.92** | **0.90** | **0.91** |
| **Mesophilic**  **α+β** | **0.83** | **0.85** | **0.83** | **0.79** | **0.84** | **0.92** |
| **Thermophilic**  **All-β** | **0.94** | **0.92** | **0.93** | **0.93** | **0.91** | **0.93** |
| **Mesophilic**  **All-β** | **0.93** | **0.91** | **0.92** | **0.94** | **0.91** | **0.96** |
| **Thermophilic**  **All-α** | **0.88** | **0.83** | **0.90** | **0.85** | **0.83** | **0.89** |
| **Mesophilic**  **All-α** | **0.90** | **0.94** | **0.94** | **0.89** | **0.93** | **0.95** |

Table S1

**Legend : This table (S1 of Supplementary Mat-S7) shows the break-up of Table-3, in the sense that it depicts the contribution for each SCOP class from Thermophilic and Mesophilic protein sets.**

**Abbreviations :**

**MFD : Mass Fractal Dimension**

**HFD : Hydrophobic Fractal Dimension**

**ROG : Radius Of Gyration**

**Hydrophobic-ROG : Hydrophobic-Radius Of Gyration**
